# An Automated Workflow to Address Proteome Complexity and the Large Search Space Problem in Proteomics and HLA-I Immunopeptidomics

**DOI:** 10.1016/j.mcpro.2025.101039

**Published:** 2025-07-21

**Authors:** Yehor Horokhovskyi, Hanna P. Roetschke, John A. Cormican, Martin Pašen, Sina Garazhian, Michele Mishto, Juliane Liepe

**Affiliations:** 1 Research Group of Quantitative and Systems Biology, Max-Planck-Institute for Multidisciplinary Sciences (MPI-NAT), Göttingen, Germany; 2 Centre for Inflammation Biology and Cancer Immunology, King's College London, London, United Kingdom; 3 Peter Gorer Department of Immunobiology, King's College London, London, United Kingdom; 4 Research Group of Molecular Immunology, Francis Crick Institute, London, United Kingdom; 5 Göttingen Graduate Center for Neurosciences, Biophysics, and Molecular Biosciences, University of Göttingen, Göttingen, Germany

## Abstract

Antigenic noncanonical epitope and novel protein discovery are research areas with therapeutical applications, predominantly done *via* mass spectrometry. The latter should rely on a well-characterized proteogenomic search space. Its size is barely known for antigenic noncanonical peptides and novel proteins, and this could impact their identification. To address these issues, we here develop an automated workflow comprised of Sequoia for the creation of RNA sequencing-informed and exhaustive sequence search spaces for various noncanonical peptide origins, and SPIsnake for pre-filtering and exploration of sequence search space before mass spectrometry searches. We apply our workflow to characterize the exact sizes of tryptic and nonspecific peptide sequence search spaces in a variety of definitions, their reduction when using RNA expression, their inflation by post-translational modifications, and the frequency of peptide sequence multimapping to different noncanonical origins. Furthermore, we explore the application of Sequoia and SPIsnake on HLA-I immunopeptidomes, thereby rescuing sensitivity in peptide identification when confronted with inflated search spaces. Taken together, Sequoia and SPIsnake pave the way for an educated development of methods addressing large-scale exhaustive proteogenomic discovery by exposing the consequences of database size inflation and ambiguity of peptide and protein sequence identification.

## Highlights

• Exact search space sizes, the effect of PTMs and the extent of peptide multimapping are characterized for proteogenomic search.
• Sequoia tool builds RNA-seq-informed and exhaustive MS search spaces.
• SPIsnake tool uses MS data to pre-filter the search spaces and improve search sensitivity.
• We show how to counteract the search space inflation in noncanonical peptide identification and overcome the associated statistical challenges.

## Graphical Abstract

**Figure:**
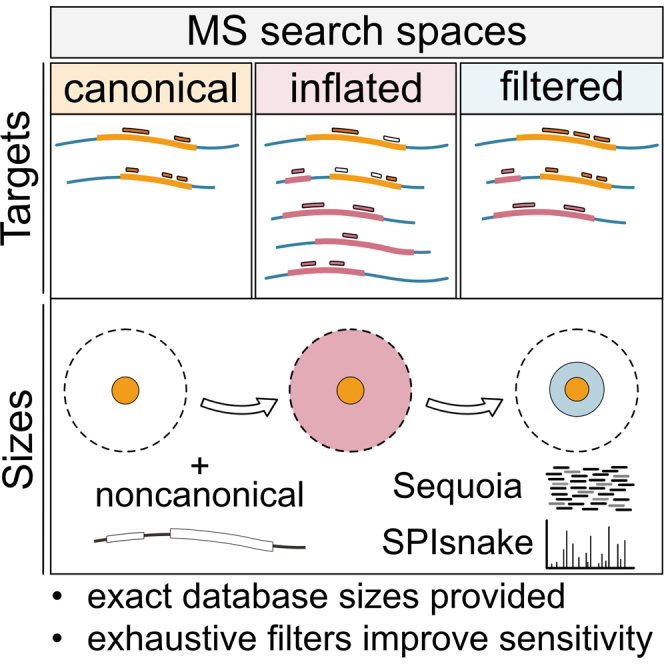

## In Brief

The exhaustive exploration of the proteogenomic search space in mass spectrometry (MS) allowed to characterize the MS databases for a variety of non-canonical protein origins and PTMs acting as useful priors for MS identification. We provide tools to inform the search spaces with RNA-seq expression and explore novel open reading frames (Sequoia) and to pre-filter the databases using the MS data (SPIsnake). Our approach increased the MS search sensitivity, showcased the discoverability challenges of noncanonical peptides, and quantified the extent of proteogenomic multimapping.

The human genome, approximately 3.1∗10^9^ base pair (bp) long, is estimated to have 62,700 genes, including 19,400 protein-coding genes (1). Alternative RNA splicing produces diverse RNA transcripts, tuning protein functionality in a cell-type-specific manner and re-shaping protein–protein interaction networks (2, 3, 4). Occurring in 95% of human multi-exon genes, alternative splicing gives rise to 89,400 protein transcripts, of which 65,500 have been validated (2, 3, 4). Additionally, noncanonical cryptic (*i.e*., putative noncoding) genomic regions like long noncoding RNA (lncRNA), pseudogenes, transposable elements, and short open reading frames (ORFs) nested in untranslated and intronic regions on primary transcripts further increase the complexity of the human proteome.

**Fig. 1:**
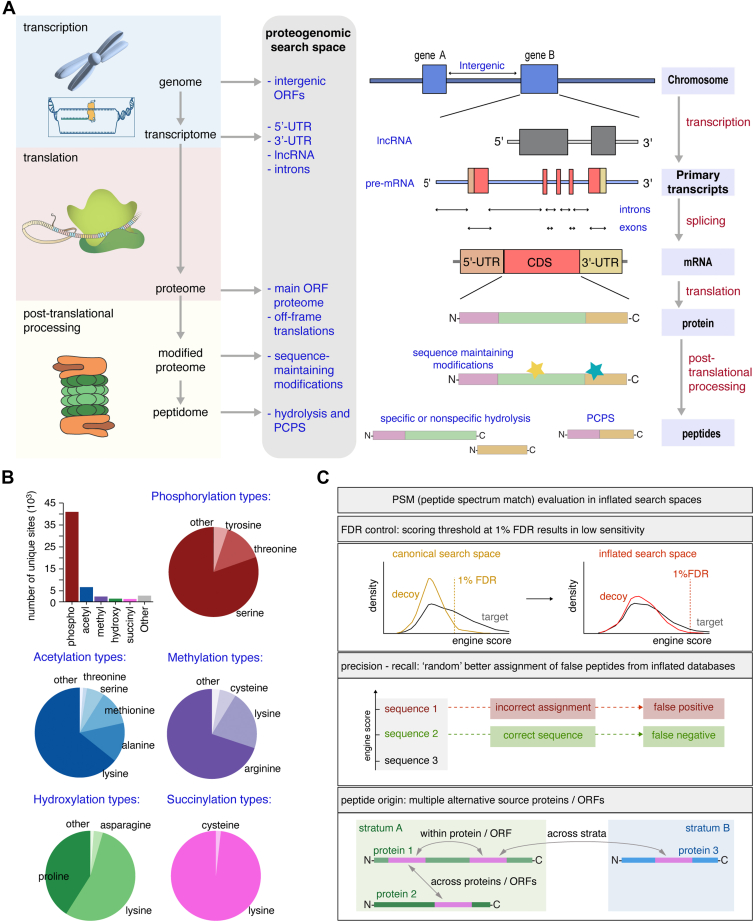
**Complexity of the proteome.***A*, proteome diversity arises from transcription, splicing, and translation of mRNA. Additional noncanonical ORFs on lncRNAs, ORFs nested in and overlapping 5′- and 3′- UTRs of transcripts, coding ORFs inside introns, and un-annotated ORFs in the intergenic regions may expand the coding potential of the genome. Noncanonical ORFs can be exhaustively defined from transcriptome and genome sequences. Post-translational modifications result in changes of amino acid residues or alter the order of amino acids in the case of proteasome-catalyzed peptide splicing. *B*, distribution of most frequent experimentally confirmed PTMs in the human proteome based on the UniProt Knowledgebase expert annotation. *C*, the large search space problem and the multimapping problem in MS. The FDR control in conditions of an increased number of high-scoring decoys results in higher score thresholds and reduced MS search sensitivity. Multiple alternative peptide origins can be found within a single ORF, across gene isoforms, and in different proteogenomic origins.

Noncanonical proteins can have different metabolic functions (5, 6, 7, 8), have a role in diversifying the antigenic landscape in Human Leucocyte Antigen class I (HLA-I) immunopeptidomes, and can be targeted by a CD8^+^ T cell response (9). HLA-I molecules typically present 8 to 15 amino acid long peptides. Defective ribosomal products (DRiPs), which are translational products that do not achieve functional integration into the proteome and are degraded within minutes in cells, as well as noncanonical peptides from genomic cryptic regions, such as intergenic and intronic sequences, also contribute to the HLA-I immunopeptidome and influence CD8^+^ T cell responses in diseases (10, 11, 12, 13, 14, 15, 16) (Fig. 1*A*). Proteasomes are the key proteases of the HLA-I antigen processing and presentation (APP) pathway (17, 18). They produce peptides of 4 to 40 amino acids in length and could cleave after every amino acid, thereby generating “nonspecific” peptides (19, 20, 21, 22, 23) and differing from other proteases like trypsin that cleave after few specific amino acids. In addition to simple cleavage by hydrolysis of polypeptides and proteins derived from noncanonical transcription and translation, proteasomes contribute to antigenic diversity by reshuffling peptide sequences during the processing of canonical proteins. Indeed, these proteases do not only cut and release peptides *via* peptide hydrolysis but also cut and paste non-contiguous peptide fragments either from the same protein—thereby generating *cis*-spliced peptides—or from two proteins—thereby generating heterologous *trans*-spliced peptides—*via* a transpeptidation reaction, named proteasome-catalyzed peptide splicing (PCPS, Fig. 1*A*) (24). PCPS is a non-random process, driven by factors only partially understood (19, 20, 23, 25, 26, 27), and occurs with a frequency considerably smaller than peptide hydrolysis (23). The location of the catalytic sites within the inner chamber of the proteasome barrel may be one of the reasons, whereby most of the known post-translationally spliced peptides are produced by proteasomes (28), although other proteases can also catalyze peptide splicing (24, 29, 30, 31, 32). Post-translational peptide splicing is not the only known post-translational modification (PTM) that alters the original antigen sequence, and many other PTMs can also enhance the antigenic variety in HLA-I immunopeptidomes (9). Indeed, the UniProt database lists over 300 confirmed chemical (sequence-maintaining) PTMs, with phosphorylation and acetylation being the most frequent (Fig. 1*B*), whereas the Unimod database includes more than 700 potential chemical PTMs (many without biological annotation). Chemical PTMs regulate protein interactions, localization, structure, function, and degradation (33, 34, 35, 36). Modified peptide recognition by T cells is evident in cancer and autoimmune diseases (37, 38, 39, 40). Proteins and peptides are often identified using mass spectrometry (MS); hence, additional to naturally occurring PTMs, technical PTMs occurring during MS sample processing also need consideration.

Standard MS protocols start with proteins that are enzymatically digested into peptides—for example, by trypsin, which mainly cleaves proteins after arginine (R) and lysin (K) (41, 42, 43), separated by chromatography, and measured with tandem MS. Peptide sequences are matched to a reference protein sequence database for identification, limiting the detection of novel proteins to those listed in the database. *In silico* approaches could be used to derive polypeptide/protein sequence databases describing novel isoforms or noncanonical translational events. In the last decade, several (proteogenomic) pipelines have been developed to identify the portion of HLA-I immunopeptidomes outside of canonical non-spliced peptides (12, 14, 15, 16, 44, 45, 46, 47, 48, 49, 50, 51, 52, 53, 54, 55, 56). However, such approaches are quickly confronted with the large search space problem, *i.e.*, the larger the sequence search space of a reference database is, the lower the identification sensitivity at low false discovery rate (FDR) (57, 58, 59). Larger databases also make it difficult to unambiguously identify peptide origins, thereby impacting protein identification accuracy (Fig. 1*C*).

While there is an intuitive understanding of the large search space problem, the impact of noncanonical peptides and PTMs on the sequence search space and the ability of successful novel peptide/protein discovery is not fully understood. By exploring and quantifying the genome's potential for diversity through noncanonical transcription, translation, and PTM, optimal methods for the identification of noncanonical peptides and proteins can be developed.

**Fig. 2:**
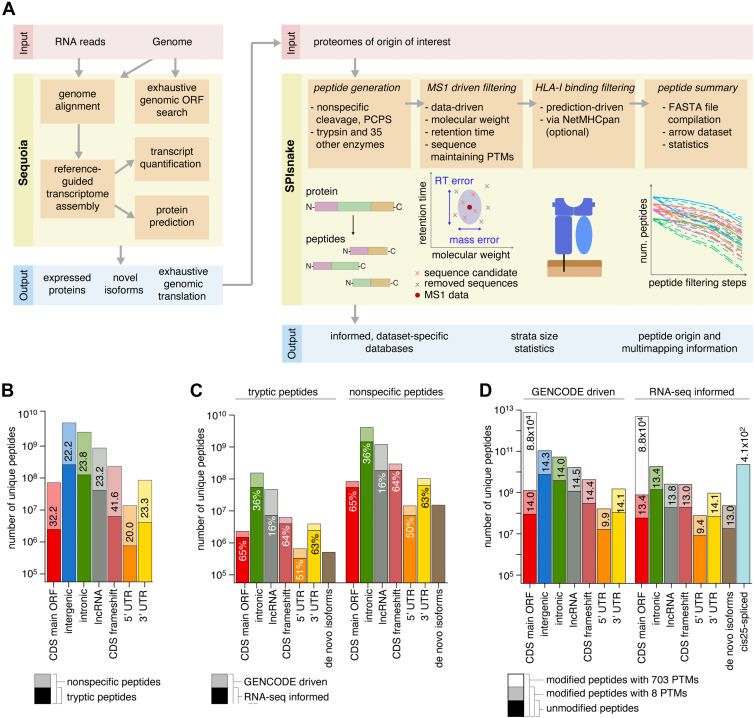
**Complexity of canonical and noncanonical proteomes and peptidomes.***A*, sequoia and SPIsnake workflow. Sequoia: Schematic of RNA-seq pipeline for informed proteogenomic strata generation. Genome and transcriptome sequences are scanned for ORFs to provide exhaustive search spaces. RNA-seq quantification refines the search space by transcript expression support and is used to search for sample-specific ORFs on de novo assembled transcripts. SPIsnake: Protein sequences are enzymatically processed *in silico* and unique peptide sequences are PTM-expanded and further filtered to keep the MW and RT that can explain the experimental data. HLA-I immunopeptidome search spaces can be additionally filtered by HLA-I-peptide binding affinity. *B*, comparison of strata sizes for tryptic and nonspecific peptides. Numbers indicate relative fold increase of nonspecific strata compared tryptic strata. No RNA-seq information was used for strata construction. *C*, strata size reduction by RNA-seq expression for tryptic and nonspecific peptides. Percentages indicate relative reduction of search space by using RNA-seq informed database compared to GENCODE driven database. *D*, impact of PTMs on peptide strata sizes. The numbers of unmodified peptides are compared to PTM-modified nonspecific peptides either with a small set of 8 common PTMs, or a set of 703 PTMs (open PTMs) or *cis25*-spliced peptide stratum. Numbers indicate relative fold increase of strata sizes by PTM combinations. The increase in *cis25*-spliced peptide numbers is relative to RNA-seq informed CDS main ORF. In (*B* and *C*), the computed tryptic peptides are 5 to 30 amino acids long, covering the typically observed length range of proteomics. Nonspecific peptides are 8 to 15 amino acids long, covering the natural length range observed in HLA-I immunopeptidomes. In (*C* and *D*) RNA-seq data from K562-A∗02:01 and K562-B∗07:02 cell lines are used.

To this end, we here propose an automated workflow comprised of Sequoia (Sequence Expression Quantification of Unknown ORF discovery and Isoform Assembly), for the creation of RNA-informed and exhaustive sequence search spaces, and SPIsnake (Spliced Peptide Identification, Search space Navigation And K-mer filtering Engine) for characterization of sequence search spaces and construction of data-driven informed search spaces prior to application in MS search engines (Fig. 2*A*).

As proof-of-concept, we carried out an exhaustive exploration of the proteogenomic search space for tryptic and nonspecific peptides derived either from known main frame coding sequences (CDS; *i.e*., canonical peptides) and off-frame CDS, or from cryptic genomic regions including lncRNA sequences, 5′-UTR, 3′-UTR, intronic and intergenic regions (Fig. 1*A* and Supplemental Table S1). Furthermore, we investigated the impact of PTMs (both chemical and PCPS) on the size of the sequence search space and quantified the multimapping problem in the context of peptide and protein identification (Fig. 1*C* and Supplemental Tables S2, and S3). We explored the application of Sequoia and SPIsnake on RNA-seq, tryptic proteomics, and HLA-I immunopeptidomics datasets, which helped to rescue sensitivity in peptide identification when confronted with inflated search spaces, and proposed database search strategies and FDR estimation approaches for stratified inflated search spaces.

## Experimental Procedures

### Mass Spectrometry

MS data of K562 cell lines’ HLA-I immunopeptidomes were collected using Orbitrap Fusion Lumos mass spectrometer coupled to an Ultimate 3000 RSLC nano pump (both from ThermoFisherScientific), as described elsewhere (57). MS data of tryptic digestions of cell proteome were measured through Thermo Scientific Orbitrap Exploris 480 mass spectrometer), as described elsewhere (58).

MS data of B721.221 HLA-I immunopeptidomes were retrieved from the ProteomeXchange Consortium *via* the PRIDE with the dataset identifier PXD025499. As described in the previous study (60), this data was collected using a Quadrupole Orbitrap mass spectrometer (Q Exactive Plus, Thermo Scientific) coupled to a nanoUPLC pump.

### RNA Sequencing (RNA-seq)

RNA was extracted from K562, K562-A∗02:01, and K562-B∗07:02 cell pellets, enriched for polyA-containing transcripts. Libraries were made using the NEBNext Ultra RNA Library Preparation Kit with random priming. Samples were sequenced on HiSeq 2 x 150 PE HO platform, resulting in a depth of 20 to 25 million reads per sample. Adapters were trimmed, and poor-quality reads were removed using Trim Galore (61) with a stringency parameter set to 5. RNA-seq dataset for B721.211 cells was retrieved from NCBI GEO GSE93315 and GSE131267.

Transcript expression was quantified with Salmon software v1.1.0 using a decoy-augmented GENCODE v33 human transcriptome with the following flags “—validateMappings –mimicBT2 –rangeFactorizationBins4 –seqBias –gcBias –reduceGCMemory –posBias –numGibbsSamples 1000”. These settings enable the bias models and selective alignment mode to improve the quantification accuracy. Additionally, k-mer length was reduced to 23 bp. 1000 samples were drawn from the resulting posterior distribution and normalized by median transcript length among gene isoforms, and then the library size (dtuScaledTPM) using the tximport R package workflow.

All the transcripts with at least 10 counts per biological replicate were considered expressed, and their corresponding proteins were used to define the RNA expression-informed mass spectrometry search space. Further details about reads trimming, quantification and data processing are described elsewhere (57).

### Cell Lines

K562-B∗07:02 and K562-A∗02:01 cell clones express single HLA-I alleles, *i.e.*, HLA-B∗07:02 and -A∗02:01, respectively. They derive from the leukemia K562 cell line (ATCCCCL-243), which does not express endogenous HLA-I and -II molecules, and their generation is described elsewhere (57). K562 clones were grown in RPMI medium with 10% FCS, 2 mM glutamin, and PenStrep.

### HLA-I Immunopeptidome Elution and Tryptic Proteome Digestion

HLA-I-bound peptides were isolated from 10^9^ cells of K562 clones through HLA-I-peptide elution using W6/32 antibody, as described elsewhere (57).

Tryptic digestions of the cell proteome obtained from the K562 cell line were carried out, as described elsewhere (58).

### Generation of GENCODE-Driven Peptide Strata

GENCODE transcriptome main annotation Release 33 (GRCh38.p13) (62) was used for TxDb object construction with R packages ‘*GenomicFeatures’* (63). Human genome “GRCh38” primary assembly from GENCODE was forged into a ‘Bsgenome’ object (64).

First, the annotated GenomicRanges were extracted for every transcript according to feature type, resulting in sequences for CDS, 5′-UTRs, 3′-UTRs, and introns. All sequences belonging to chromosomes but outside the gene regions were classified as intergenic. Coordinates of lncRNA were extracted from the GTF transcriptome annotation file. Next, for every feature, all longest open reading frames (ORFs) per stop codon starting with ATG, CTG, GTG, ATC, ACG, and at least 8 amino acid-long have been identified with the “ORFik” R library (65).

The resulting genomic coordinates were checked for overlap with coding sequences (CDS) and were grouped into three groups: “doesn’t overlap with CDS”, “overlaps with CDS, frameshifted”, and “overlaps with CDS, in-frame”. The corresponding nucleotide sequences were extracted and translated. Fuzzy codons that can be translated non-ambiguously to an amino acid or to ∗ (stop codon) were translated. Ambiguous fuzzy codons were translated to X.

The CDS *in silico* translation does not always result in exactly the GENCODE reference translation sequence, including selenocysteine and codon readthrough events and other manual curation refinements (see GENCODE transcriptome tags).

Generalized Levenshtein (edit) distance was estimated between every reference protein and 6 frame translations of the parent transcript; the frame with the least distance was tagged as a main ORF.

The genes shared between the human X chromosome and Y pseudo-autosomal region are annotated twice in GENCODE; we removed their duplicates as well as any annotated protein sequence shorter than 5 amino acids (Fig. 2*A*).

An RNA-informed database was constructed using the same procedure but only applied to the set of expressed transcripts.

### Sequoia

We implemented a computational platform (Sequoia, Sequence Expression Quantification with Unknown ORF discovery and Isoform Assembly) for RNA-seq analysis to inform search space generation. The pipeline uses the genome and transcriptome annotation together with RNA-seq reads to derive expressed and exhaustive ORFs. First, the reads in FASTQ format are trimmed from adapters and low-quality baes using fastp (66). Next, Salmon in selective alignment mode is used to quantify the expressed transcripts while using the genome as a decoy (67). The following parameters are used: --validateMappings, --mimicBT2, --rangeFactorizationBins 4, --seqBias, --gcBias, --reduceGCMemory, --posBias. The estimated transcript quantities are processed with the tximport (68) R package, transcript abundancies are derived with “dtuScaledTPM” and gene expression—with “lengthScaledTPM” method, which normalizes the abundancies by library depth and gene/transcript length and was designed for differential expression quantification and to avoid the target length bias. Proteins originating from the expressed transcripts are saved in a FASTA file.

The second capability of Sequoia is the derivation of an exhaustive search space, as described in the “Generation of GENCODE-driven peptide strata” section above.

In addition to reference transcriptome quantification, reference-guided transcriptome assembly can be performed to discover new transcripts not present in the reference transcriptome. First, the genome sequence is indexed and aligned to the genome using the STAR (69) aligner in a two-pass mode. The novel splice junctions discovered in the first alignment round with at least 3 uniquely mapped fragments per sample are collected across all samples and considered in the second alignment pass.

The resulting BAM files are filtered by Phred score to keep the reads with an average base quality above 20 for further processing. Reference-guided transcript assembly with Stringtie (70) is done for each input sample in a conservative mode with the minimal transcript length of 200 bp. The resulting GTF files with transcript coordinates of novel isoforms are merged with Stringtie merge using the following flags: -c 0 -m 50 -T 0 -f 0.05. In addition, gffcompare tool (71) is applied to match the assembled transcripts to the reference transcriptome annotation using the parameters: -D -V -A -X -K.

An annotation of a union transcript set across samples is passed to the gffread (71) together with the genome sequence, to cluster and validate the transcripts and extract their nucleotide sequences. The following options are used by gffread: -F -P --adj-stop --no-pseudo --force-exons --keep-genes --keep-comments --merge -K -Q -Y --t-adopt -v -E -T.

A subset of transcripts without exact matches of intron chain, *i.e.*, all the new transcripts, are formatted into gff3 format by Transdecoder (72) and undergo further ORF discovery. First, the Transdecoder LongOrfs module identifies the best ORF per transcript that’s at least 100 AA long, and protein domains are searched with Hmmer3 (73) tool using Pfam (74) database as a reference. The Transdecoder Predict module is next applied with default parameters to classify the ORFs as coding/noncoding, considering the domain identifications from the previous step and keeping only a single best ORF per transcript. Next, the ORFs are aligned against the genome and amino acid translation sequences are extracted using Transdecoder utils: cdna_alignment_orf_to_genome_orf.pl and gff3_file_to_proteins.pl.

In addition, the *de novo* expanded transcriptome is indexed and quantified with Salmon-tximport workflow as described above. A set of expressed reference transcript ORFs and ORFs from novel transcripts comprises an expanded proteogenomic search space that can be used for further MS searches.

### Gene Fusion Identification

We adapted the Arriba pipeline (75) for the fusion gene identification from the K562 cell line RNA-seq and ran it with default parameters. All the fusions with at least one medium- or high-confidence identifications were kept for further investigation, and the following fusion types were dropped: head-to-tail 5′-5′ and 3′-3′, non-translatable fusions, fusions with unaltered CDS, potential transcription readthrough events, fusions where the stop codon is upstream of the fusion junction, and non-predictable CDS. We compared the pairs of fusion genes with the DepMap and FGDB2 databases (76, 77).

### Computational Implementation of Exhaustive Framework: SPIsnake

The exhaustive framework (SPIsnake, Spliced Peptide Identification, Search space Navigation, and K-mer filtering Engine) described here is implemented in Snakemake (78) and available as readily executable, user-friendly tool on GitHub (https://github.com/QuantSysBio/SPIsnake).

The speed and efficiency of SPIsnake rely on parallelization and the availability of computational resources in a high-performance computing cluster. SPIsnake can per default be run within Slurm (79), which is a popular HPC workload manager and which allows the user to control computational resources, such as CPUs, memory, and the distribution of jobs to different compute nodes.

The SPIsnake pipeline is implemented in Snakemake (78) workflow management system and uses a custom Docker (80) container with all software dependencies, including NetMHCpan. All data processing was performed in *R* (81) unless specified otherwise.

The workflow uses three user inputs:

1. A Master table that specifies all the proteomes to be used, which peptide lengths to generate, and whether to generate variable PTMs.
2. An Experiment design is a table that contains information about which datasets of observed MWs to use, what the MS-1 error ranges (in ppm) are, which fixed modifications to use, and whether or not to predict IC50 with NetMHCpan
3. Additional parameters are specified in features. yaml configuration file

First, all the proteomes specified in the Master table are indexed using samtools (82) and clustered using mmseqs linclust (83) with the following parameters: “mmseqs easy-linclust -e 1.000E-03 --spaced-kmer-mode 1 --spaced-kmer-pattern 110,101”.

A combination of FASTA index and a table with protein-cluster assignment is used to read in only the proteins that belong to a given sequence similarity cluster using random access to a FASTA file. This behavior is especially helpful when working with translated genome databases that can take up to several GB of volume.

A user-defined size constant is used to split the input proteomes into the chunks of fixed size, that contain proteins assigned to the same sequence identity clusters as estimated by linclust (83). Proteins with similar sequences that belong to the same clusters will be processed together to reduce the peptide duplication. Proteins that are longer than the maximum length parameter are split into sub-sequences with an overlap of double the size of maximum intervening sequence length. *Biostrings*, *stringr* and *stringi* packages (84, 85, 86) are used for working with fasta files and string manipulation here and throughout the pipeline.

Next, all the peptide generation jobs are defined: a separate process will be started for every unique combination of a proteome chunk, hydrolysis/splicing rule, intervening sequence length, and peptide length. This is done to control the RAM build-up due to R global string pull and potentially large size RAM consumption by PTM generation on long peptide sequences and compression-decompression costs when saving arrow datasets.

In order to speed up the generation of proteasome-spliced peptide sequences, an index of substring positions is pre-computed. Specifically, for every peptide length and max intervening sequence length, a generation of spliced peptide sequences is represented as two substring operations that use the N-/C-terminal coordinates of splice reactants. These 4 coordinates are computed for every protein length in the range specified by the user.

Next, the predictor for RT in chromatography is trained, and the RT error is estimated as described below in the section “Peptide filtering by RT”. Following that, peptide generation and filtering jobs are executed. One job per node first generates the peptides and filters them initially by MW, next by both MW and RT. For peptides between 8 and 15 amino acids in length, NetMHCpan can be used to predict the IC_50_ and filter the peptides according to a user-specified threshold.

Data filtering and manipulation are done with the *data.table* (87) R library and a combination of *dplyr*-*tidyr* frontend with *dtplyr* backend (88, 89, 90) to translate tidy R expressions into fast data.table code. Multithreading is achieved by a combination of native *data*.*table* multithreading and the default R “mcmapply” function from the *parallel* package for vectorized functions. Non-vectorized functions are parallelized *via* “mclapply” function. All the apply-functions are using a fork cluster to create new working processes. A parallelly (91) R package is used to register a finalizer for the fork cluster to stop the cluster when the garbage is collected.

All the peptide-protein mapping tables and information about peptides, their predicted properties, and whether or not they pass the filters for each of the datasets are saved as *arrow* datasets (92) in parquet format with “lz4” compression.

Once all peptides have been generated and filtered, a unique fasta file is generated per biological group—this unique set of peptides is the desired pipeline output to be used for proteomic search on a set of samples that share the proteogenomic search space. If necessary, the statistics of proteogenomic database sizes are computed using the arrow datasets produced earlier with *dplyr* interface.

### Computation of Post-translationally *cis*-Spliced Peptides

For a protein of a given length, a maximum intervening sequence length, and output peptide length, two sets of peptide sequences can be derived.

First, a set of non-spliced peptides can be derived by moving a sliding window of length N along the protein sequence. This implemented by creating an “Integer Ranges List” class object with start and end positions defined *via* vectorized of base R “seq.default” function applied to create the abovementioned set of sliding window coordinates. The sequence strings are obtained by using the “extractAt” function that extracts the substrings at the given positions from a protein sequence in a fast and vectorized way.

Second, the positions for the first and second splice reactants of forward and reverse cis-spliced peptides of the longest protein are computed, such that the distance between splice reactants does not exceed the maximum intervening sequence length. This matrix is computed once for the longest protein in the input and is filtered to exclude all the indices with coordinates ranging outside the shorter protein length, The sequences of spliced peptides are derived in two steps: the splice reactants are extracted the same way as non-spliced peptides and are concatenated pair-wise using the “stri_join” function from the ”*stringi*” R library (86). Once the peptide sequences have been generated, they are converted to the “data.table” class table to be used downstream.

Since the maximum intervening sequence length is a user parameter, spliced peptides can be generated for any maximum intervening sequence length besides the default value of 25.

### Peptide Filtering by MW

For peptides without PTMs or with PTMs that are not specific to C-/N- termini, a fast MW computation using *Biostrings* (84) R package is made. A peptide is converted into an “AAStringSet” class, and a “letterFrequency” matrix is multiplied by the diagonal matrix of monoisotopic masses for a given amino acid or its modified form. For fixed modifications with modifications specific to C-/N- termini, a slower method of MW estimation first checks that the peptide C-/N- termini contain the amino acid that should carry a PTM, and a sum of all mass differences associated with all PTMs is computed.

Filtering by MW is done using the non-equi join with “inrange” *data.table* function, that checks whether any of the computed MWs of peptide sequences is within the intervals corresponding to detected molecular weights, given the MS1-error. The detected MW is derived from the m/z ratio and the precursor charge state, to perform matching on a single MW instead of multiple possible m/z values per sequence.

When performing a search with 8 common PTMs, the following modifications were used: peptide N-terminal acetylation, carbamidomethylation of cysteine (C), deamidation of asparagine and glutamine (N, Q), oxidation of methionine (M), phosphorylation of serine, threonine, and tyrosine (S, T, Y). Analysis of the K562-cell line data was performed with 5 ppm MS1-error.

### Peptide Filtering by RT

RT in chromatography can be learnt from peptide sequences for a given experiment. A regular proteomic search allows identification of canonical peptides at 1% FDR, and these peptides are used to calibrate the RT predictor and estimate the prediction error (see “Database search for RT calibration” for details). Here, the standard MS identifications are used to train predictors of chromatographic retention time from amino acid composition, and then predict RT for the MW-filtered search space.

Two methods for RT prediction are available: an additive achrom model as implemented in *pyteomics* package (93) and a deep neural network (AutoRT) (94). A cross-fold validation based on the unique peptide sequence split is used to estimate the RT prediction error. A user-defined quantile threshold (here set to 0.99 quantile) is used to set an absolute prediction error cut-off and its mean value across folds is used as a RT prediction error.

Peptides that pass the MW filter are additionally filtered by both MW and predicted RT, using the provided *data.table* non-equi join such that predicted MW and RT belong to the range of values that could explain both the observed MWs and RTs in the data.

In the *pyteomics* implementation, *reticulate* (95) package is used to communicate between R and Python. In this framework, peptides that carry amino acids not observed during training will be excluded from further analysis since it’s impossible to estimate the weights for amino acids that were not present in the training set. Peptides carrying fixed PTMs undergo filtering by both MW and RT, while the variable modifications are filtered only by MW. Since the RT prediction for unseen or infrequent PTMs is not reliable, no further filters are applied to peptides that carry them.

### Database Searches for RT Calibration

For analysis of the K562 and B721.221 cell lines in this study, PEAKS DB searches of the canonical immunopeptidome were required for RT calibration in SPIsnake and further used for analysis of predicted binding affinity (IC_50_) distributions. Database searches were carried out on the canonical RNA-seq-informed reference database containing 43,578 protein entries for the K562 cell line and 64,159 protein entries for the B721.221 cell line. Precursor mass tolerance was set to 5 ppm, and MS/MS tolerance was set to 0.02 Da for the K562 datasets. Precursor mass tolerance was set to 10 ppm, and MS/MS tolerance was set to 0.05 Da for the B721.221 datasets. No post-translational modifications were set (neither variable nor fixed).

PEAKS DB was selected for these initial searches as opposed to MSFragger, which was used for later searches of expanded search spaces due to previous benchmarking showing PEAKS DB as the most performant search engine for canonical immunopeptidomics (96, 97).

### Peptide Filtering by HLA-I-Peptide Binding Affinity Predictions

For non-spliced and spliced peptides from HLA-I immunopeptidomes, an additional filtering layer can be used—*i.e*., the prediction of HLA-I-peptide-binding affinity to only keep the peptides that passed a user-defined IC_50_ threshold. NetMHCpan4.1 was used for HLA-I-peptide binding affinity prediction with the binding affinity data (“-BA” option). For K562 HLA-A∗02:01 and HLA-B∗02:07, an IC_50_ threshold of 5000 nM was used, retaining 92.8% 8-15mers and 96.2% 9-11mers. A PEAKS DB search against the canonical proteome in the B721.221 dataset resulted in poor predicted IC_50_ distributions for detected canonical peptides longer than 11 amino acids, pointing towards unspecific peptide elution. Only 8% of 12 to 15-amino acid-long peptides surpassed the 5,000 nM threshold in the B721.221 dataset. Hence, we restricted our analysis to 8 to 11-amino acid-long peptides and used the same IC_50_ cutoff of 5000 nM as in K562, resulting in 92% of 8 to 11-amino acid-long peptides surpassing the threshold.

### Peptide Aggregation, Uniqueness, and Strata Statistics

To estimate the numbers of unique peptides and other search space statistics, a *dplyr* query to arrow dataset is used *via dbplyr* API (98). This allows for fast filtering and aggregation of processed data, since only the arrow backend is used without loading the data onto R memory. Column-oriented parquet storage format allows to selectively load only the subset of columns to be used for further computations and the hive partitioning structure is split by enzyme rule, intervening sequence length, proteome, amino acid prefix of the peptide and peptide length to perform summarization on the relevant sub-sets of data only. Partitioning the peptides by the first 1 (tryptic cleavage) or 2 (nonspecific and PCPS) amino acids of the sequence allows to selectively retrieve distinct subsets of the search space for downstream.

Larger-than-RAM data are processed by passing arrow dataset queries to *duckdb* (99) with a temporary directory on the disk. By default, the sizes of unfiltered search spaces are reported for each combination of proteome, enzyme digestion rule, peptide length, and intervening sequence length (for PCPS). In addition, the sizes of search spaces after data-driven filters can be provided for every group of input masses and RTs.

### Peptide Multimapping Analysis Across Origins

The arrow databases of peptide-protein mapping information, as well as the SPIsnake data-driven stratum pre-filtering, were used to investigate the peptide mapping across alternative origins. To process the large volume of peptides, the data were batched corresponding to SPIsnake output partitioning by stratum, peptide length and prefix, and enzymatic digestion rule. Arrow interface to parquet files was used for disk operations (92), the processing was implemented *via* tidy dbplyr interface to duckdb SQL database and R in-memory data.table backend (87, 88, 89, 98, 99). Estimating the strata overlaps upon SPIsnake filtering involves an additional step of filtering the peptides that could be supported by MS data per replicate. Average values across replicates were used for figure generation.

Gene-ORF mapping for the CDS main ORF was retrieved from the GENCODE annotation. Multimapping across ORFs and genes within strata was done by first counting the number of distinct origins per peptide, followed by counting the number of peptides per each number of origins. Multimapping between strata pairs was comprised of counting the number of distinct peptides in each stratum and the number of shared peptides. The ratios of set intersection to each of the strata sizes were used to represent multimapping between strata pairs.

### Analysis of Peptide Identification in HLA-I Immunopeptidomes by Varying Sequence Reference Databases

To study the impact of database size and RT filtering on peptide identification, we performed a database search on the 3 MS files for the K562-B∗07:02 cell clones and 3 MS files for the B721.221 B∗07:02 cell clones with the MSFragger search engine using databases of varying sizes. MSFragger was used for this benchmarking as opposed to PEAKS DB due to its ability to handle extremely large search spaces (100) and previous use in searching cryptic immunopeptidomes (101).

MSFragger outputs were then recorded *via* Percolator. Features considered by Percolator were the MSFragger score of the PSM, the difference between that PSMs score and the score of the second ranked PSM for the same spectrum, the sequence length, the charge state of the peptide, and the mass difference between the theoretically expected and experimentally measured peptide mass. These databases were generated by applying SPIsnake to the canonical proteome (filtered by RNA-seq support) and the cryptic proteome (containing the canonical proteome (CDS main ORF), CDS frameshift, 5′ UTR, 3′ UTR, long noncoding RNA, and intronic strata). In both cases, the databases were based on GENCODE v33. We did not consider PTM-peptides for this pilot study. Furthermore, due to concerns over non-HLA-I-bound peptides present in the data, the sequence length was restricted to 8–11mers for the B721.221 cell line.

All canonical and cryptic strata were informed by our RNA-seq data, and we further informed the search space to different extents with SPIsnake pre-filtering.

For both the canonical and the noncanonical, we extracted (i) all peptides which were MW-filtered by precursor masses in the MS data, (ii) all peptides which were MW-filtered and which passed the SPIsnake RT filter, and (iii) all peptides which were MW-filtered and which passed the SPIsnake RT and binding affinity filters. Hence, 6 sequence databases were generated per cell line, the canonical proteome with all MW-filtered peptides, the canonical proteome with all MW and RT-filtered peptides, the cryptic proteome with all MW-filtered peptides, and the cryptic proteome with all MW and RT-filtered peptides. To ensure a fair decoy strategy, we then reversed the canonical and noncanonical reference databases and performed the same filtering steps on the reversed sequences to generate 6 corresponding decoy databases.

FASTA files were created for each database with a protein entry for each peptide, and MSFragger search was then performed with no enzymatic cleavage (hence, missed cleavages were not relevant). Thus, the database sizes for the K562 data were 20,750,651 (MW filtered canonical), 7,195,599 (MW-RT filtered canonical), and 298,669 (MW-RT-HLA-I binding filtered canonical), 628,403,789 (MW filtered expanded), 180,523,229 (MW-RT filtered expanded), and 8,953,992 (MW-RT-HLA-I binding filtered expanded) peptide sequences. For the B721.221 data, the database sizes were 15,853,128 (MW filtered canonical), 7,539,374 (MW-RT filtered canonical), and 319,344 (MW-RT-HLA-I binding filtered canonical), 628,403,789 (MW filtered expanded), 115,779,041 (MW-RT filtered expanded), and 5,729,603 (MW-RT-HLA-I binding filtered expanded) peptide sequences.

Precursor mass tolerance was set to 5 ppm, and MS/MS tolerance was set to 0.02 Da for the K562 datasets. Precursor mass tolerance was set to 10 ppm, and MS/MS tolerance was set to 0.05 Da for the B721.221 datasets. No post-translational modifications were set (neither variable nor fixed).

PSM and peptide identifications were filtered based on FDR estimation to analyze both PSM/peptide yield and the distribution of spectral angles (compared to Prosit MS2 spectra prediction) for the identifications at FDR between 0.1% and 5%. A peptide-level FDR of 1% was applied to perform further analysis of spectral angle analysis. Peptides containing unmodified cysteines were not considered for spectral angle analysis as carbamidomethylation of cysteine was a fixed modification in the original Prosit training data (102). Prosit prediction and spectral angle computation were performed using inSPIRE version 1.5 (97, 103).

### Computation of Search Space for Peptides Allowing Common PTMs

To investigate the inflating effects of common PTMs on the search space, SPIsnake was executed using oxidation of methionine, carbamidomethylation of cysteine, N-terminus acetylation, deamidation of asparagine and glutamine, and phosphorylation of serine, threonine, and tyrosine as variable PTMs for MW-filtering. Tryptic PTMs were generated for the full proteomes, nonspecific peptide expansion with PTMs has been performed on samples of the original proteome. The length distribution of each proteome was split into 10 equal intervals and up to 50 entries were sampled without replacement from each interval, resulting in 300 to 500 proteins per FASTA file. Sampling of intergenic sequences was deeper—up to 500 ORFs, resulting in 2404 sequences.

After SPIsnake generated all PTM combinations and performed MW-filtering, aggregate statistics were computed to derive the total number of PTMs considering and ignoring the peptide-PTM isomers, before and after MW-filtering for each peptide separately and for all the peptides in the search space. In case the original search space exceeded 5∗10^6^ peptides, a random sample of 5∗10^6^ peptides was drawn for further aggregation. The ratios of PTM-expanded to unmodified sequence search space sizes were computed for each proteome and peptide length separately and used to predict the sizes of PTM-expanded full search spaces by multiplying the ratios by the corresponding exhaustive unmodified sizes.

Computation of search space for *cis*-spliced peptides with varying maximum intervening sequence length, as well as for canonical peptides, allowing 703 PTMs

We used a reference proteome length distribution to sample 98 proteins and generate *cis*-spliced peptides with the maximum intervening sequence length of 25, 50, 100, and 200 amino acids. The proteome was sampled to cover the whole range of protein length (in amino acids). We obtained the number of unique *cis*-spliced peptides for each maximum intervening sequence length and each filtering step for each of the 98 proteins.

The same sampled reference proteome of 98 proteins was used to explore in the same manner the search space of canonical peptides, allowing all 703 PTMs as possible variable modifications. All combinations of PTMs with a maximum of 2 variable PTMs per peptide were generated, and the per-protein and total stratum numbers were computed for unmodified and MW-filtered PTM-peptides. The ratios of PTM-expanded to unmodified sequence search spaces were computed for each peptide length and used to predict the sizes of PTM-expanded full search spaces by multiplying the ratios by the corresponding exhaustive unmodified sizes.

### Calculation of Theoretical Number of Unmodified, Nonspecific Spliced, and Non-Spliced Peptides

We derived analytical solutions to calculate the number of spliced and non-spliced peptides that could theoretically be derived from a given protein. The number X of non-spliced peptides of length N that could theoretically arise from a substrate of length L is:

$$X_{non-spliced}=\left\{ \begin{array}{c} L-N+1\;\\ 1\; \end{array}\right.\begin{array}{c} \forall \;L>N\\ \forall \;L=N \end{array}$$

To derive the positions of all spliced peptides, we define four indices i,j,k and n that denote the start and end position of the first (i,j) and second (k,n) splice-reactant, respectively. The corresponding number of peptides is calculated by summing over interval ranges that form valid spliced peptides. *Cis*-spliced peptides can be formed *i* forward or reverse ligation. The number of all forward *cis*-spliced peptides of length N that could theoretically arise from a substrate of length L is:

$$X_{fwdCis}=\sum_{i=1}^{L-N}\;\sum_{j=i+L_{ext}-1}^{N-L_{ext}+i-1}\;\sum_{k=j+2}^{L-N+j-i+2}1=\frac{1}{2}\left(N-2L_{ext}+1\right)\left(L-N\right)\left(L-N+1\right)$$

$L_{ext}$ denotes the minimal splice-reactant length and is set to *1* per default. Similarly, the number of theoretically possible forward *cis*-spliced peptides with an intervening sequence length restriction $I_{\max}$ is:

$$X_{\max intervening,fwdCis}=-\frac{1}{2}I_{\max}\left(N-2L_{ext}+1\right)\left(I_{\max}-2L+2N-1\right)$$

The intervening sequence length is calculated as the distance between j and k for forward *cis*-spliced peptides.

Analogously, the number of theoretically possible reverse *cis*-spliced peptides is calculated as:

$$X_{revCis}=\sum_{k=1}^{L-N+1}\;\sum_{n=k+L_{ext}-1}^{N-L_{ext}+k-1}\;\sum_{i=j+1}^{L-N+n-k+2}1=\frac{1}{2}\left(N-2L_{ext}+1\right)\left(L-N+1\right)\left(L-N+2\right)$$

$$X_{\max intervening,revCis}=\left(N-2L_{ext}+1\right)\left(I_{\max}+1\right)\left(1-\frac{1}{2}I_{\max}+L-N\right)$$

The intervening sequence length is calculated as the distance between n and i for reverse *cis*-spliced peptides.

Finally, combining the numbers of forward and reverse *cis-*spliced peptides and adjusting for cases where the upper intervening sequence length restriction $I_{\max}$ exceeds the protein length, the following approach was taken:

$$X_{cis-spliced}=\left\{ \begin{array}{c} -\frac{1}{2}I_{\max}\left(N-2L_{ext}+1\right)\left(I_{\max}-2L+2N-1\right)+\left(N-2L_{ext}+1\right)\left(I_{\max}+1\right)\left(1-\frac{1}{2}I_{\max}+L-N\right)\\ \forall \;I_{\max}\geq L-N\;\\ \frac{1}{2}\left(N-2L_{ext}+1\right)\left(L-N\right)\left(L-N+1\right)+\frac{1}{2}\left(N-2L_{ext}+1\right)\left(L-N+1\right)\left(L-N+2\right)\\ \forall \;I_{\max}\;<\;L-N \end{array}\right.$$

To calculate the number of theoretical *trans*-spliced peptides between two proteins of length $L_{1}$ and $L_{2}$ the following approach was taken:

$${SR1}_{\min}=\max\left\{L_{ext};\;N-L_{2}\right\}$$

$${SR1}_{\max}=\min\left\{L_{1};\;N-L_{ext}\right\}$$

$$x=\sum_{{SR1=SR1}_{\min}}^{{SR1}_{\max}}\left(L_{1}-SR1+1\right)\left(L_{2}-N+SR1+1\right)=-\frac{1}{6}\left(1+{SR1}_{\max}-{SR1}_{\min}\right)\left(-6-6L_{1}-6L_{2}-6L_{1}L_{2}+{SR1}_{\max}-3L_{1}{SR1}_{\max}+3L_{2}{SR1}_{\max}+2{SR1}_{\max}^{2}-{SR1}_{\min}-3L_{1}{SR1}_{\min}+3L_{2}{SR1}_{\min}+2{SR1}_{\max}{SR1}_{\min}+2{SR1}_{\min}^{2}+6N+6L_{1}N-3{SR1}_{\max}N-3{SR1}_{\min}N\right)$$

$$X_{trans-spliced}=\max\left(0;x\right)$$

The number of non-spliced peptides was computed on all RNA-seq-informed and uninformed strata. *Cis*- and *trans*-spliced peptide numbers were calculated only for the RNA-seq-informed CDS main frame reference database.

### Computation of Upper Bound for Expected Number of Unique *Cis* and *Trans*-Spliced Peptides

Computation was based on an assumption that every splicing event independently samples with replacement from a population of all possible sequences of a specific length that can be generated by shuffling 20 amino acids. The formula for the number of unique sequences was:

$$num\_unique=20^{N}\left(1-{\left(1-\frac{1}{20^{N}}\right)}^{M}\right)$$

where N stands for the peptide length and M for the number of splicing events. It holds true for both *cis* and *trans*-spliced peptides. It takes into account redundancy caused by the finiteness of the sampling space, but does not take into account redundancy caused by the specificity of splicing events, amino acid distribution in proteins, and similarity between proteins. Therefore, it is only an upper bound, not an estimate of the number of unique peptides. Computations were done using the Python library *SymPy* (104) v1.12 to combat numerical problems.

### Statistical Analysis

Computational and statistical analysis has been implemented in *R* v4.1.1 (105), except for analysis presented in the result section “Towards a strategy for improved sensitivity and FDR estimation for noncanonical peptide identification in HLA-I immunopeptidomics by applying Sequoia and SPIsnake” and for computation of expected unique post-translationally spliced peptides presented in the result section’ “SPIsnake, post-translationally spliced peptide search space and sequence uniqueness per protein”, where *python* v3.9.11 was used instead.

### Software

Sequoia is available on GitHub under https://github.com/QuantSysBio/sequoia

SPIsnake is available on GitHub under https://github.com/QuantSysBio/SPIsnake

Analyses were carried out in *R* v4.1.1. and *Python* v3.9.11.

Figures have been generated in *R* or in *Python* v3.9.11 using the *Plotly* library (106) and postprocessing was done with Adobe Illustrator v26.1 and 29.5.

MS analysis was carried out with MSFragger v3.7.0.

Rescoring of MSFragger outputs was performed with Percolator v3.05.

## Results

### Defining the Size of Sequence Search Spaces of Canonical and Noncanonical Peptide Strata by Sequoia and SPIsnake

To characterize the canonical and noncanonical sequence search space, we developed and applied Sequoia to generate ORF databases from various (non)canonical strata. Sequoia performs exhaustive genomic ORF search, genomic alignment of RNA reads, reference-guided transcriptome assembly (to discover novel transcripts) and quantification, as well as protein prediction (Fig. 2*A*, Supplemental Table S1 and Supplemental Methods). The resulting ORF databases were processed with our second tool, SPIsnake, a stand-alone parallelized tool that computes all theoretically possible peptides with user-defined specificities (*e.g.,* tryptic peptides, nonspecific peptides) and length restrictions for all peptide strata provided by Sequoia (Fig. 2*A*, Supplemental Fig. S1, Supplemental Table S1 and Supplemental Methods). Hence, Sequoia and SPIsnake allowed us to characterize the sequence search space, which we defined as the number of unique peptides derived from a stratum to be explored in an MS analysis.

It is theorized that a cell population expresses approximately 10,000 proteins at a given time, which, upon *in vitro* trypsin digestion, could roughly result in 100,000 peptides with a K or R at their C-terminus (107). At the same time, the human genome’s CDS main ORF gives rise to 65,500 proteins, from which we computed 2.8∗10^6^ unique tryptic peptides 5 to 30 residue long (this length restriction is due to MS technical restrictions in peptide identification), therefore implying that in any given tryptic proteome analysis, at most 3.5% of all theoretical tryptic peptides are discoverable in an analyte (Fig. 2*B*). From the same human genome’s CDS main ORF, we computed that there were 9.2∗10^7^ theoretical nonspecific peptides 8 to 15 amino acids long, *i.e.,* peptides that could be generated upon cleavage of every amino acid by less stringent proteases such as proteasomes and presented by HLA-I complexes. This corresponded to a 32-fold increase in the sequence search space compared to tryptic peptides (Fig. 2*B*). Due to the difference in the target length distribution of tryptic and HLA-I peptide search spaces, we also investigated the effect of cleavage specificity, as such, in the overlapping 8 to 15 residue long range. The R/K frequency among all amino acids of the CDS main ORF is 11.5%, which corresponds to a 76-fold decrease compared to the nonspecific cleavage, if the peptide length restrictions and amino acid order of protein sequences were neglected, and R/K are assumed to be at the termini. Our exhaustive estimate using SPIsnake, comparing the exact numbers of unique peptides in the 8- to 15-residue long range, resulted in a 68- to 94-fold difference to nonspecific cleavage, allowing us to separate the compositional and amino acid sequence order contributions within protein sequences (Supplemental Fig. S2 and Suppelemental Table S3).

It has been estimated that a cell presents up to 30,000 unique peptides on their HLA-I complexes at a given time (108), which is reduced to an average of 5000 unique peptides in HLA-I immunopeptidomes eluted and measured by so far commonly used MS methods. Therefore, we estimated that in an average HLA-I immunopeptidome sample at most 0.005% of all theoretical peptides in the canonical sequence search space were discoverable, which represented a 651-fold decrease of discoverable peptides in immunopeptidomics compared to tryptic proteomics analysis, and well highlighted the increased challenge of nonspecific peptide identification compared to tryptic proteomics. Similarly, noncanonical nonspecific peptide strata were between 20- to 42-fold larger than the corresponding tryptic peptide strata. The latter overall consisted of 5.2∗10^8^ unique tryptic peptides, 183-fold larger than the canonical CDS main ORF-derived tryptic peptide stratum, thereby illustrating the challenge of noncanonical peptide and protein identification (Fig. 2*B* and Supplemental Table S4). In this comparison, we should bear in mind that we do not know how many of the noncanonical *in silico* computed protein and polypeptide sequences were actually transcribed and translated and, therefore, in theory discoverable. If we assumed that noncanonical proteins comprised at most 10% of detectable canonical proteins, we could estimate that 0.002% of the theoretical noncanonical tryptic peptides were discoverable, which represented a 1833-fold decrease compared to the 3.5% of the discoverable canonical tryptic peptides, thereby exasperating the challenge of noncanonical protein identification.

To tackle this large search space problem in proteomics and immunopeptidomics, one strategy is to narrow down the search space by incorporating extra information, such as considering transcript abundances derived from RNA-seq experiments (14, 58, 97, 109, 110, 111, 112, 113, 114). Therefore, to better characterize the large search space problem related to noncanonical proteomics and immunopeptidomics, and the information content carried by RNA-seq data, we analyzed a multi-omics dataset of K562 cell line clones (*i.e*., RNA-seq, tryptic proteomics and HLA-I immunopeptidomics) to investigate the sample-specific information content provided by different omics.

Within Sequoia, the K562 cell line’s de novo augmented transcriptome was quantified (Fig. 2*A*). To identify transcript-derived protein or polypeptide sequences most likely to be present in the analyte of a given biological replicate, we excluded all transcripts with fewer than 10 reads, as computed by the dtuScaledTPM of tximport (68). Employing RNA-seq data, we observed a reduction of the search space of canonical and noncanonical peptide strata to 65% and 16 to 64%, respectively, compared to the full GENCODE-driven search space. The search space was equally reduced among tryptic and nonspecific peptides (Fig. 2*C* and Supplemental Table S4). Similar search space reduction has been reported to result from other additional sequencing information, such as ribosome profiling (115).

RNA-seq could not only provide a reduced MS search space with additional support for expressed transcripts but also be used to expand the search space by utilizing data-driven transcriptome assembly for novel peptide and protein discovery. Within Sequoia, we made use of Stringtie2 (70) reference-guided transcriptome assembly and discovered 8659 novel transcripts originating from 5353 genes in the K562-derived RNA-seq data, not annotated in GENCODE v33 (62). ORF prediction resulted in 5626 distinct ORFs, with the majority coming from multi-exon transcripts with at least one junction match, intron retention events, or containing the reference, indicating that most of the observed novel transcript ORFs were alternative splicing products. In addition, 85 ORFs coming from 78 nonannotated genomic loci were identified (Supplemental Table S5), resulting in 1.8∗10^7^ unique peptides from the *de novo* isoforms nonspecific peptide search space (Fig. 2*C* and Supplemental Table S4).

Gene fusions resulting from chromosomal rearrangements are a known hallmark of cancer cells, driving the malignant transformation and utilized as a biomarker. To address this, we applied the Arriba gene-fusion detection pipeline (75) to the K562 cell line’s RNA-seq data (Supplemental Table S4). Initially, we identified 125 fusions from 108 gene pairs detected in 1 or more samples with at least one medium or high-quality breakpoint prediction, which were filtered to exclude non-translatable fusions, fusions with unaltered CDS and potential transcription readthrough events resulting in 25 breakpoints from 18 gene pairs and 6 more intragenic breakpoints from 5 genes. We compared the resulting non-intragenic fusion gene pair list to the fusions reported in the literature and found support for 10/18 gene pairs: 7 in the FGB2 database across all samples and 7 in K562-specific database from the DepMap. Four detected fusions were supported by both databases, including the BCR-ABL1 fusion, a signature of the K562 cell line (76, 77, 116). If all filtered 31 breakpoints were true and only gave rise to unique novel peptides, this would result in 2.8∗10^3^ novel nonspecific 8 to 15 residue long peptides. This number is small in comparison to the other strata in consideration, therefore we didn’t follow up on this extension of the search space, even though in a biomarker discovery scenario, the proteogenomic identification of such peptides would certainly be beneficial.

### Dissecting the Impact of PTMs on the Large Search Space Problem by Applying SPIsnake

Utilizing RNA-seq data within Sequoia could reduce the sequence search space, thereby facilitating the analysis of the human proteome and HLA-I immunopeptidome with PTMs. We initially investigated the impact of commonly considered chemical PTMs in tryptic proteomics and nonspecific immunopeptidomics on the sequence search space by applying SPIsnake and exhaustively computing all possible modified tryptic and nonspecific peptide forms from RNA-seq informed and GENCODE-driven strata. We considered M oxidation, N/Q deamidation, C carbamidomethylation, S/T/Y phosphorylation, and N-terminal acetylation as 8 variable modifications; *i.e.*, they may or may not occur on the peptide’s amino acid residues. To note, fixed modifications, such as C carbamidomethylation in tryptic proteomics, do not inflate the search space, but change the m/z fragmentation pattern and, hence, were not further investigated here. Allowing those 8 variable amino acid-specific PTMs, and a maximum of two modifications per peptide in MS analysis of CDS main ORF, created on average 20 modified forms for tryptic peptides and 13 modified forms for nonspecific peptides, in a peptide length-dependent manner (Supplemental Fig. S3, *A*–*D*). We computed a 9 to 14-fold larger search space for canonical and noncanonical nonspecific peptide strata with the 8 commonly used PTMs compared to the cognate unmodified peptide strata. The search space of nonspecific CDS main ORF-derived peptides with the 8 commonly used PTMs was similar to or larger than most of the nonspecific noncanonical unmodified peptide strata (Fig. 2*D* and Supplemental Table S6).

As for noncanonical proteins and peptides, the frequency and occurrence of PTMs in proteomics and immunopeptidomics is a matter of debate, in part due to technical challenges. The eight commonly used PTMs investigated here represent a rather small selection of all PTMs that have been described in literature so far. To investigate all possible PTMs so called “open” MS searches have been developed and used to verify PSM assignment to other noncanonical peptide sequences (46, 51, 117). By using a sampling approach with SPIsnake (see Methods), we computed the number of peptide forms that could theoretically be observed when allowing maximum two PTMs per peptide out of a catalogue of 703 PTMs (Suppelemental Table S5), which was the full list of PTMs approved by the Unimod team (118). On average, 8.8∗10^4^ modified forms per peptide were observed (Supplemental Fig. S3*E* and Supplemental Table S6). Therefore, by applying this figure to unique peptides derived from RNA-seq informed CDS main ORF, we obtained approximately 5∗10^12^ nonspecific modified peptide forms (Fig. 2*D* and Supplemental Table S6).

PCPS, a type of sequence-altering PTM, has been investigated within HLA-I immunopeptidomes through exhaustive database and *de novo* search strategies. Previous research focused on *cis*-spliced peptides with limited intervening sequence lengths (47, 48), inspired by an *in cellula* study on a single *cis*-spliced peptide (119). To address the large search space problem in exhaustive searches, SPIsnake was used to calculate all possible *cis-*spliced peptides with up to 25 amino acids intervening sequence length (as applied in (48)), termed *cis25-*spliced peptides. This resulted in the computation of 2.4∗10^10^ unique nonspecific *cis25*-spliced peptides from RNA-seq-informed CDS main ORF. This search space was 408 times larger than that of unmodified nonspecific peptides and 30 times larger than that of peptides modified with 8 common PTMs from the same stratum (Fig. 2*D*). The search space for *cis25*-spliced peptides was double than all combined noncanonical peptide strata, although yet 216-fold smaller than the search space for PTM-peptides from CDS main ORF considering 703 variable PTMs.

Thus, depending on their definitions, sequence-maintaining PTMs may have a greater impact on the search space than *cis25*-spliced peptides, especially in “open” MS searches for PTM analysis (Fig. 2*D*).

### Increasing Information Content in Large Search Spaces of Peptide Sequences by Applying SPIsnake

In MS, search engine performance is not only affected by the size of the sequence search space, but also by the ratio of true positive sequences over all peptide sequences in a reference database, *i.e.*, the percentage of discoverable peptides in an analyte. Accordingly, the information content of the sequence search space has been shown to impact search engine performance (57). These ratios can be optimized by considering other peptide features such as peptides’ molecular weights (MWs) and MS chromatography retention times (RTs). So far, the latter has been mainly used for a more accurate scoring of PSMs after search engine application (23, 97, 120, 121, 122, 123). Conversely, given a conventional MS search output based on the canonical peptide search space (Supplemental Fig. S1*C*), SPIsnake can make use of peptides’ MWs and RTs as well as dataset-specific trained RT predictors to reduce the expanded sequence search space prior to search engine application. SPIsnake can allow the inclusion of fixed and variable PTMs into theoretical peptide computation and filtering. For HLA-I immunopeptidome datasets, SPIsnake can employ HLA-I-peptide binding affinity predictors (*i.e.*, NetMHCpan-BA (124)) to further inform the sequence search spaces through a maximum predicted IC_50_ threshold (Fig. 2*A*, Supplemental Fig. S1*C*, and Supplemental Table S2).

**Fig. 3:**
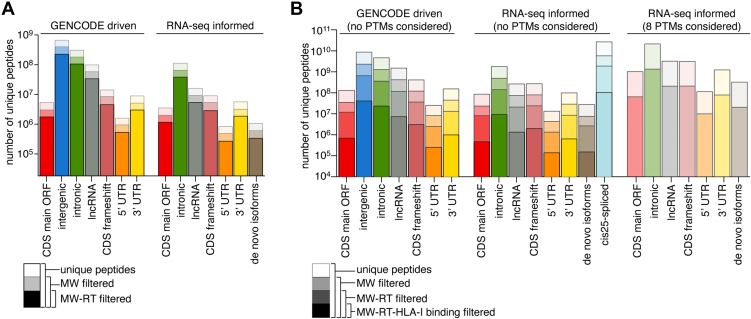
**Proteome complexity in MS.***A* and *B*, impact of MS1 characteristics on sequence search space for (*A*) tryptic and (*B*) nonspecific peptides. Shown are numbers of unique peptides per stratum, which is either unfiltered, filtered based on molecular weight (MW filtered), filtered by molecular weight and retention time (MW-RT filtered), or additionally filtered by HLA-I-peptide binding affinity predictions (MW-RT-HLA-I binding filtered). In (*A* and *B*) analysis represents the mean over 2 biological replicates, measured in one or two technical replicates, respectively, derived from K562-B∗07:02 HLA-I immunopeptidome.

To investigate this alternative strategy, we applied SPIsnake (see Experimental Procedures and Fig. 2*A*) to tryptic proteomics and HLA-I immunopeptidomics dataset derived from K562 cell lines based on Sequoia constructed GENCODE-driven and RNA-seq-informed canonical and noncanonical peptide strata. By applying the MW and RT information contained in the tryptic proteome dataset using SPIsnake, we could reduce the search space approximately 3-fold for both canonical and noncanonical peptide strata, regardless of RNA-seq information (Fig. 3*A* and Supplemental Table S7). By applying SPIsnake to RNA-seq informed HLA-I immunopeptidomics datasets derived from K562-B∗07:02 (Fig. 3*B* and Supplemental Table S8) and -A∗02:01 (Supplemental Fig. S4*A* and Supplemental Table S7) cell lines, we obtained a 12-fold and additional 14-fold decrease in sequence search space by applying the MW-RT filter and the additional HLA-I-peptide binding affinity filter, respectively, corresponding to an overall sequence search space reduction of 140- to 200-fold across strata and datasets. A similar reduction was observed when considering GENCODE-driven strata (Fig. 3*B*, Supplemental Fig. S4*A*, and Supplemental Table S8). Therefore, upon employing SPIsnake the search space of noncanonical peptides in HLA-I immunopeptidomes was only 1.4-fold larger than canonical peptides in HLA-I immunopeptidomes without employing SPIsnake (Fig. 3*B* and Supplemental Fig. S4*A*).

By applying MW-RT and HLA-I-peptide-binding affinity filters of SPIsnake on RNA-seq informed HLA-I immunopeptidomes, the sequence search space of *cis25*-spliced peptides was reduced on average 180-fold, thereby being reduced to the double of the canonical peptides without applying SPIsnake (Fig. 3*B*, Supplemental Fig. S4*A*, and Supplemental Table S8). Therefore, by employing SPIsnake, we could increase the rate of discoverable canonical peptides in HLA-I immunopeptidomes from 0.005% to 0.82%, which was only 4-fold smaller than the rate of discoverable tryptic canonical peptides, and of noncanonical peptides from 4∗10^-6^ to 8∗10^-4^%, which was only 7-fold smaller than the rate of discoverable canonical peptides in HLA-I immunopeptidomes without employing SPIsnake.

To note, within SPIsnake, the IC_50_ threshold of the HLA-I binding affinity filter must be carefully chosen. In the K562 data set, we set the threshold of 5,000 nM, which corresponded approximately to the 95%-ile of the IC_50_ distribution of identified canonical peptides when employing PEAKS DB search engine at 1% FDR (Supplemental Fig. S5 and Supplemental Table S8).

An important difference between tryptic peptides and HLA-I immunopeptidome peptides is their length. Tryptic peptides range from 5 to 30 amino acids, while HLA-I peptides are usually 8 to 15 amino acids long, with 9 amino acids being the most common. Since peptide length affects information content and MS search engine performance, we further investigated its impact on sequence search spaces using SPIsnake. Upon application of the MW-RT-HLA-I-peptide-binding affinity filter, the search space was most considerably reduced for 15 amino acid long peptides (averaging a 7762-fold decrease), whereas the least reduction was observed for 9 amino acid long peptides (averaging a 47-fold decrease), across all investigated peptide strata (Supplemental Fig. S4, *B*–*E* and Supplemental Table S7).

### SPIsnake Post-Translationally Spliced Peptide Search Space and Sequence Uniqueness Per Protein

**Fig. 4:**
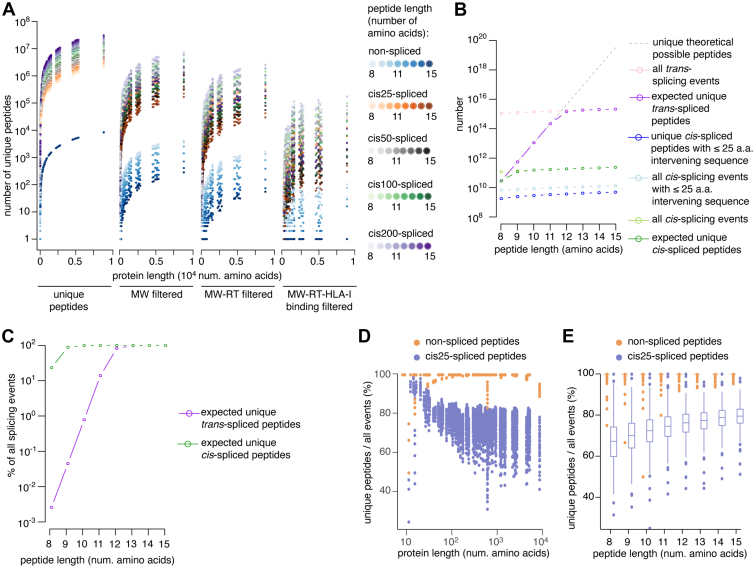
**Immunopeptidome complexity and proteasome-catalyzed peptide splicing.***A*, impact of MS1 characteristics on sequence search space for post-translationally *cis*-spliced peptides considering a range of intervening sequence length. Shown are numbers of unique *cis-*spliced peptides per protein, peptide length and maximum intervening sequence length, which are either unfiltered, filtered based on molecular weight (MW filtered), additionally filtered by RT prediction (MW-RT filtered), or additionally filtered by HLA-I-peptide binding affinity predictions (MW-RT-HLA-I binding filtered). Analysis represents the mean over 2 biological replicates, measured in one or two technical replicates, respectively, derived from K562-B∗07:02 HLA immunopeptidome. *B* and *C*, the numbers of *cis*- and *trans*-spliced peptides compared to the theoretical upper boundaries. Numbers of peptide sequences, number of splicing events and expected number of unique peptides, as well as an upper boundary of 20 amino acid combinations (equivalent to the *de novo* search space) (*B*) and fraction of expected unique peptides to number of splicing events (*C*) are reported per peptide length. The number of all *cis*- and *trans*-splicing events and the expected number of unique *cis*- and *trans*-spliced peptides were calculated without intervening sequence length restriction and determined theoretically (see Experimental Procedures), and hence, present an upper bound. *D* and *E*, ratio of number of unique peptide sequences to the number of possible peptide hydrolysis/splicing events on per-protein basis (*D*), or aggregated by peptide length (*E*). Analysis in (*A*, *D*, and *E*) represents the mean over 2 biological replicates of K562-A∗02:01 and K562-B∗07:02 immunopeptidomes.

In the preceding analysis, the PCPS sequence search space was limited to *cis25*-spliced peptides, *i.e., cis*-spliced peptides with an intervening sequence not longer than 25 residues. This restriction might catch only part of the *cis*-spliced peptides in HLA-I immunopeptidomes, bearing in mind the recent results on *cis*-spliced peptides produced by proteasomes while processing entire proteins *in vitro* (23), where the median of 79 residues was observed. Therefore, by applying SPI snake in a sampling approach to the K562 HLA-I immunopeptidome datasets (see Experimental Procedures), we investigated the impact of the intervening sequence length restriction on the sequence search space of *cis*-spliced peptides. The increase in the maximum intervening sequence length from 25 to 200 amino acids increased the sequence search space by 5- to 6-fold, depending on peptide length, and was equally affected by SPIsnake data-driven filters (Fig. 4*A* and Supplemental Table S9).

To expand the study to post-translationally *trans*-spliced peptides, we derived mathematical expressions to compute the number of theoretically possible post-translationally *cis*- and *trans*-splicing events and the number of expected unique post-translationally *cis*- and *trans*-spliced peptides without intervening sequence length restriction (Fig. 4, *B*–*E* and Supplemental Fig. S6). The number of theoretically possible splicing events is an upper bound for the number of unique peptides since various splicing events could generate identical peptide sequences. A more precise estimate was defined by calculating the expected number of unique spliced peptides, which was up to 4.2-fold smaller for *cis*-spliced and up to 40,000-fold smaller for *trans*-spliced peptides compared to all theoretically possible *cis-* and *trans-*splicing events, respectively (Fig. 4, *B* and *C* and Supplemental Table S9). However, the calculation of the expected number of unique spliced peptides did not consider the specificity of splicing events and amino acid frequencies in proteins (see Experimental Procedures), which would further decrease the estimate. Among 8 to 15 amino acid long peptides, we computed 1.2∗10^12^ expected unique *cis*-spliced peptides (50-fold larger than *cis25*-spliced peptides), and 7.2∗10^15^ expected unique *trans*-peptide sequences (Fig. 4*B* and Supplemental Table S9).

Since the sequence search space for post-translationally spliced peptides was the largest stratum without considering chemical PTMs, we preliminarily investigated the potential problem of multimapping within the same protein, *i.e*., assigning a peptide to its precise location within an ORF (Fig. 1*C*). To this end, we compared the number of unique nonspecific peptide sequences derived from example ORFs of varying length to the theoretical number of peptide events derived from the same ORFs ignoring sequence composition. The number of unique *cis25-*spliced peptide sequences ranged from 40% to 80% of all theoretical possible *cis25-*splicing events for ORFs longer than 100 amino acids (Fig. 4*D*). This was particularly pronounced for shorter peptide sequences (Fig. 4*E*). Contrary, non-spliced peptides derived from the same ORF could be assigned with high certainty to a location within that ORF, with only a few exceptions for shorter peptide sequences (Fig. 4, *D* and *E*).

### Quantifying the Peptide Origin Ambiguity in Proteomics and Immunopeptidomics with SPIsnake

**Fig. 5:**
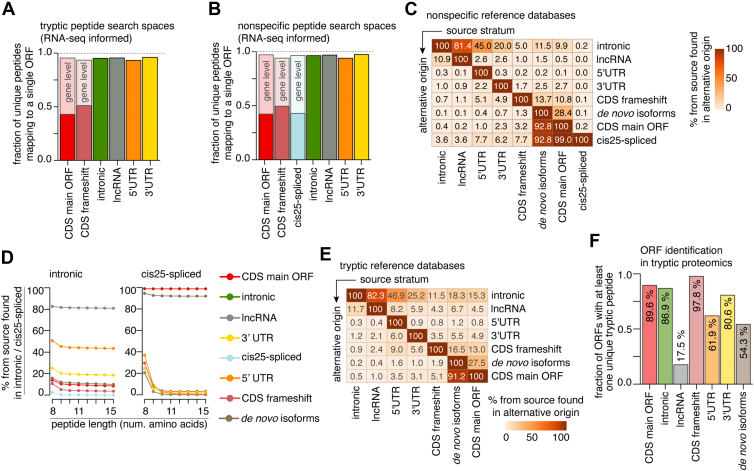
**Where do I come from?—The problem of finding the correct peptide origin.***A* and *B*, multimapping within strata for tryptic (*A*) and nonspecific (*B*) sequences derived from RNA-seq informed strata. Shown is multimapping across ORFs, gene-level multimapping also shown for CDS main ORF, CDS off-frame and *cis25*-spliced peptides. *C–F*, Multimapping across RNA-seq informed strata for nonspecific (*C* and *D*) and tryptic (*E–G*) peptides. In (*C* and *E*), for each source stratum, the fraction of peptides that multimap to an alternative stratum (alternative origin) is displayed for nonspecific (*C*) and tryptic (*E*) peptides. *D*, percentage of nonspecific peptides derived from RNA-seq-informed strata found in intronic regions and among *cis25*-spliced peptides on peptide length for RNA-seq-informed strata. *F*, fraction of ORFs of a given RNA-seq-informed stratum that have at least one unique tryptic peptide that does not map to any other RNA-seq-informed strata, thereby indicating the fraction of ORFs that can be identified unambiguously with tryptic proteomics. Analysis in (*A–E*) represents the mean over 2 biological replicates of K562-A∗02:01 and K562-B∗07:02 immunopeptidomes.

Although the above described “multimapping problem”, which is specific to multimapping within a given protein (or ORF), was mainly relevant for post-translationally spliced peptides, other kinds of multimapping problems could be related to other noncanonical strata. For instance, a given peptide sequence could originate from multiple proteins belonging to the same stratum. Our exhaustive analysis with SPIsnake could assess all potential sources of peptides from a given stratum for both tryptic and nonspecific peptides. We found that only 42% and 50% of non-spliced peptides derived from CDS main ORF and CDS frameshift, respectively, did not multimap within its stratum. In contrast, 93 to 97% of the other cryptic peptides strata did not multimap within its stratum, regardless of whether we consider either tryptic or nonspecific peptides (Fig. 5, *A* and *B* and Supplemental Table S10). The extensive multimapping of canonical peptides could be explained by the presence of protein isoforms because only 3% of the canonical peptides could be mapped to multiple genes. For *cis25*-spliced peptides, 57% could originate from multiple ORFs within the same gene, but only 4% from more than one gene. Neither expression filtering with RNA-seq data nor the application of SPIsnake to reduce the search space and inform resulting databases appeared to have a clear impact on the multimapping-problem within strata (Fig. 5, *A* and *B*, Supplemental Fig. S7, *A*–*C*, and Supplemental Table S10). Comparing all theoretical peptide events to uniquely derived nonspecific peptide sequences through our exhaustive SPIsnake approach corroborated these results (Supplemental Fig. S7, *D* and *E*, Supplemental Tables S10, and S11).

Further investigating the multimapping problem, we explored the extent of peptide sequence multimapping across canonical and noncanonical peptide strata using SPIsnake (Fig. 5, *C* and *F*, Supplemental Fig. S7, *F* and *G*, and Supplemental Table S12). We observed that nonspecific peptides from all strata frequently did multimap to intronic regions (Fig. 5*C*). 99% of *cis25*-spliced peptides were unique and not found in other strata, and only 3% to 8% of noncanonical cryptic strata overlapped with *cis25*-spliced peptides. In contrast, nearly all canonical peptide sequences (99%) could also be *cis25*-spliced peptides (Fig. 5*C*). All methods for *cis*-spliced peptide identification did and should always consider these peptide sequences as canonical CDS main ORF peptides. In addition, *de novo* isoform peptides had a 93% overlap with CDS main ORF and *cis25*-spliced peptides (Fig. 5*C*).

Using SPIsnake to create MW-RT-HLA-I-binding filtered peptide strata increased the multimapping of both canonical and noncanonical peptides to intronic regions and *cis25*-spliced peptides, although varying by dataset and HLA-I haplotype (Supplemental Fig. S7*F*). Shorter peptides were more likely to multimap to intronic regions and *cis25*-spliced peptides, particularly for 8 amino acid peptides (Fig. 5*D*).

Overall, tryptic peptides from RNA-seq informed peptide strata show a higher tendency to multimap than nonspecific peptides, particularly to intronic regions and lncRNA (Fig. 5*E* and Supplemental Fig. S7*G*). This observation is crucial for the precise identification of noncanonical ORFs in tryptic proteomics. To address this aspect, we applied SPIsnake to the RNA-seq informed dataset and computed the fraction of ORFs with at least one unique tryptic peptide among the peptide strata. The protein/polypeptides derived from lncRNA and *de novo* isoforms had the smallest fraction of unique tryptic peptides (18% and 54%, respectively), hence, significantly limiting their unambiguous identification (Fig. 5*F*).

Towards a strategy for improved sensitivity and FDR estimation for noncanonical peptide identification in HLA-I immunopeptidomics by applying Sequoia and SPIsnake.

As proof-of-principle, we investigated the effect of SPIsnake-informed database construction, and hence the effect of increasing the percent of discoverable peptides, on the performance of the MSFragger search engine in the identification of canonical and noncanonical peptides in the HLA-I immunopeptidome from 2 cell lines: (i) the above described K562-B∗07:02 cell line, and (ii) the B721.221-B∗07:02 cell line (60) (Supplemental Table S13).

**Fig. 6:**
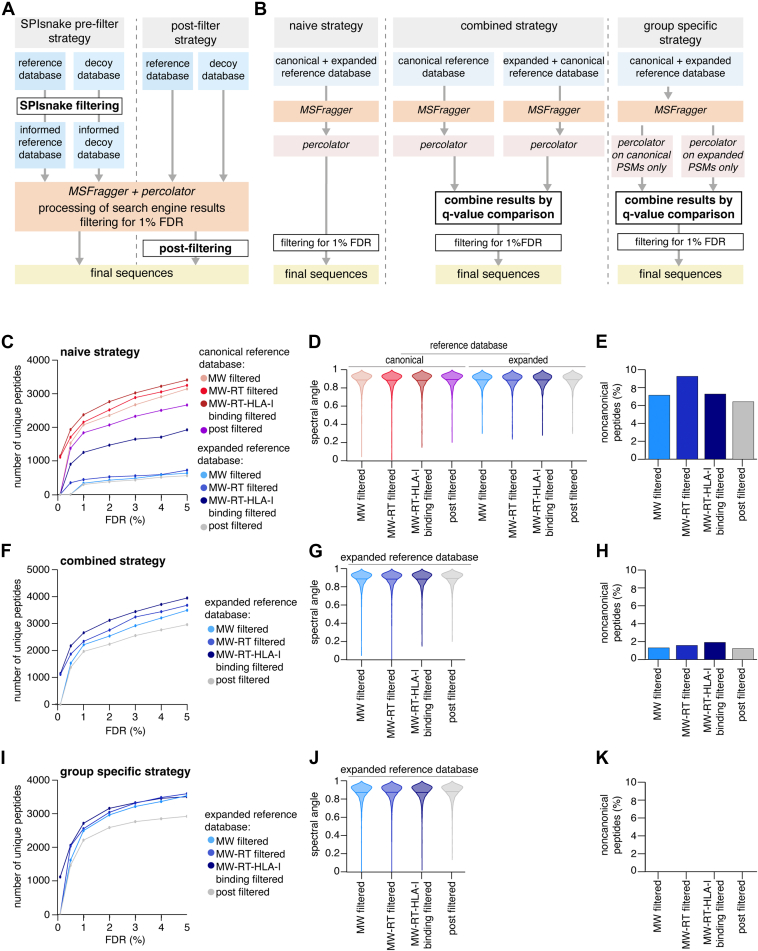
**Strategies for noncanonical peptide identification.***A*, illustration of pre- and post-filtering approaches. *B*, schematic of naïve and combined strategies for identification of (non)canonical peptides. The combined strategy prioritizes canonical peptides over noncanonical peptides in a probabilistic manner while the group specific strategy performs completely separate FDR estimation. *C–K*, MSFragger search engine and Percolator rescoring performance tested on K562-B∗07-02 cell line immunopeptidomes, when confronted with a canonical reference database (RNA-informed CDS main ORF) or an expanded reference database (multiple RNA-seq informed strata together). Databases were either pre-filtered by MW-RT or pre- and post-filtered by MW-RT-HLA-I binding. Number of identified peptides at given FDRs (*C*, *F*, and *I*), spectral angle distributions for PSMs of all peptides identified at 1% FDR (*D*, *G*, and *J*) and percentages of noncanonical peptides identified at 1% FDR (*E*, *H*, and *K*) using the naïve (*C–E*), combined (*F–H*), or group specific (*I–K*) strategy.

By applying Sequoia to RNA-seq data from both cell lines, we generated a small RNA-seq informed reference database of CDS main ORFs (‘canonical reference database’) and a greatly expanded reference database including several noncanonical peptide strata (‘expanded reference database’, see Methods for full details). For this pilot analysis we excluded both sequence altering (*e.g*., PCPS) and sequence maintaining (*e.g*., chemical) PTMs. All canonical and cryptic strata were informed by our RNA-seq data and we further informed the search space to different extents with SPIsnake pre-filtering (Fig. 6*A*). FDR estimation was achieved *via* target-decoy approach, whereby the canonical or expanded reference database was reversed. The decoy database for each target database was generated by performing the corresponding filtering of the reversed reference databases using SPIsnake. Both data sets showed similar strata sizes between target and decoy databases generated (Supplemental Fig. S8, *A* and *B*), indicating that we were not biasing the target decoy approach with the pre-filtering strategy. Hence, this target-decoy strategy ensured similar structure and information content in the target and decoy database, thereby allowing robust FDR estimation. The MS data was searched using these databases with MSFragger and rescored *via* Percolator (see Experimental Procedures for full details).

Furthermore, we also benchmarked a post-filter strategy by applying the RT and HLA-I-peptide binding prediction cut-offs after MS2 spectrum assignment with MSFragger searching Sequoia’s RNA-seq informed reference databases to the same K562-B∗07:02 cell line and B721.221-B∗07:02 immunopeptidome datasets (Fig. 6*A*). Such post-filter strategies have been utilized by others to tackle the lack of precision in large search spaces (12, 15, 27, 44, 51, 56, 125, 126, 127, 128, 129, 130). Performance of MSFragger was measured by using the standard metric of unique peptides and PSMs identified at estimated 1 to 5% FDR thresholds, as well as by assessing the quality of PSMs *via* the distribution of spectral angles between experimental spectra and the Prosit-predicted spectra (102) for the cognate peptides (see Experimental Procedures). To benchmark these different approaches, we tested the performance of 3 differing identification strategies for noncanonical peptide discovery (Fig. 6*B*).

To establish a baseline, we began with a naive strategy (Fig. 6, *C* and *E* and Supplemental Fig. S9, *A*–*C*), performing MSFragger searches against the Sequoia generated canonical and expanded reference databases upon applying the three possible SPIsnake filters (Suppelemental Tables S14–S18). MSFragger searches using a MW-filtered database is a standard strategy as most common search engines also filter reference databases by MW prior to MS2 spectrum matching. Hence, the MW-filtered approach represented the reference against which other methods were judged. Naïve approach immediately illustrated the large search space problem, with the distributions of target and decoy PSM scores becoming harder to distinguish in the larger search space in the K562-B∗07:02 cell line and B721.221 B∗07:02 immunopeptidome datasets (Supplemental Fig. S8, *C*–*H*), which we further investigated in the analysis reported in Figure 6, *C*–*K* and Supplemental Figs. S9–S11. The Naive approach yielded 2072 unique peptides and 4846 PSMs identified at 1% FDR (Fig. 6*C* and Supplemental Fig. S9*A*) for the K562 dataset. For the B721.221 dataset 1331 unique peptides and 4330 PSMs were identified at 1% FDR (Supplemental Figs. S10*A* and S11*A*). Switching from the canonical to the expanded reference database reduced peptide identification by 69% to 76% (Fig. 6*C* and Supplemental Fig. S10*A*). Employing SPIsnake pre-filtering increased the number of identified peptides, particularly with the MW-RT-HLA-I binding filter that partially compensated the drop of peptide identification upon expansion of the reference database and increased peptide identification by 14 to 15% for the canonical database compared to the standard MW-filter (reference) strategy and by 47 to 107% for the expanded database compared to the reference. This supports the effectiveness of SPIsnake pre-filtering in managing large search spaces, in contrast to a post-filtering strategy, which also increased accuracy through RT and HLA-I binding information but decreased peptide identifications (Fig. 6*C*, Supplemental Figs. S9*A*, S10*A*, and S11*A*).

Across filtering approaches, identification rates—defined as number of peptides identified at 1% FDR divided by the database size—were correlated with the percent of discoverable peptides (Supplemental Fig. S9*B* and S11*B*). Spectral angle distribution was consistent among strategies (Fig. 6*D* and Supplemental Fig. S10*B*). The percentage of noncanonical peptide identifications was estimated at between 7.1% and 9.3% *via* the reference and pre-filtering approaches and slightly lower estimated of 6.4 to 6.6% for the post-filtering approach (Fig. 6*E* and Supplemental Fig. S10*C*). Lower average spectral angle distribution among noncanonical peptides was observed for all methods (Supplemental Figs. S9*C*, and S11*C*).

To better identify noncanonical peptides, we explored a combined strategy (Fig. 6, *B*, *F*–*H*, Supplemental Figs. S9, *D*–*F*, S10, *D*–*F*, S11, *D*–*F*, and Supplemental Table S19). This method involved merging the search results from the canonical and expanded databases, selecting the MS2 spectrum assignments with the lowest q-value. The smaller size of the canonical database should provide fewer decoy candidates, since the extended database contains both canonical and novel targets, leading to more confident assignments and lower q-values in the canonical database. Consequently, this should allow for the preferential selection of canonical peptides over similar noncanonical peptides that may score just marginally higher. This combined strategy using the expanded reference database significantly enhanced peptide identification compared to the naive strategy, maintaining the peptide yield similar to the levels of the canonical strategy (Fig. 6, *C* and *F*, and Supplemental Fig. S10, *A* and *D*), although still being able to explore noncanonical strata. The combined strategy confirmed the benefit of the MW-RT-HLA-I binding filter (Fig. 6*F* and Supplemental Fig. S10*D*), maintained high-quality MS2 spectrum matches (Fig. 6*G* and Supplemental Fig. S10*E*) and provided a more cautious estimate of noncanonical peptide frequency at 1 to 3% (Fig. 6*H* and Supplemental Fig. S10*F*), compared to the naïve strategy (Fig. 6*E* and Supplemental Fig. S10*C*).

Finally, we applied a most stringent approach, *i.e*., a group specific FDR estimation (Fig. 6B,I-K). In this approach, while the expanded and canonical database were searched together in MSFragger, the PSMs were separately processed *via* Percolator. This made the identification of peptides from the expanded reference even more challenging since the ratio of correct peptides in the expanded reference is even smaller (Supplemental Figs. S9, *B*, *E*, and *G*, S10, *B*, *E*, and *G*, S11, *B*, *E*, *G*, and *H*, and Supplemental Table S20). This strategy yielded less peptides and PSMs then the combined approach (Fig. 6*I* and, Supplemental Fig. S10*G*) with a similar spectral angle distribution (Fig. 6*J* and Supplemental Fig. S9*H*). Furthermore, with this strategy and a 1% FDR threshold, no noncanonical peptides were identifiable in the K562-B∗07:02 (Fig. 6*K*) and B721.221-B∗07:02 (Supplemental Fig. S10*I*) immunopeptidome datasets. Hence, when using this strategy would require a more powerful search engine, or accepting noncanonical peptides identified at a lower confidence threshold.

In summary, SPIsnake's filters enhanced peptide identification at 1% FDR across various downstream identification methods. Different downstream approaches gave varying estimates of the frequency of noncanonical peptides in the analyte, with lower estimates and slight increases in the quality of noncanonical PSMs identified (Supplemental Fig. S12). With any downstream approach, the use of the SPIsnake RT and HLA-I binding filtered database yielded the highest peptide recall (Fig. 6, *C*, *F*, and *I* and Supplemental Fig. S9, *A*, *D*, and *G*) with similar spectral quality metrics across methods (Fig. 6, *D*, *G*, and *J*, Supplemental Fig. S9, *C* and *F*, S10, *B*, *E*, and *H*, and S11, *C*, and *F*).

The heightened sensitivity allowed for stricter FDR thresholds, maintaining high identification rates. For instance, a combined strategy with MSFragger + Percolator at 0.5% FDR using SPIsnake's pre-filtered database led to a 16 to 22% rise in peptide identification compared to a 1% FDR post-filter strategy, enabling more stringent FDR limits while ensuring accurate FDR estimations, as opposed to the post-filter strategy where no common approach exists for estimating the actual FDR after filtering.

## Discussion

The sequence content of proteogenomic databases acts as an informed prior about the sample composition, but if the prior assumptions are incorrect, peptide and protein identification can be affected. In this study, we confirmed that excessive database inflation undermines FDR control and increases multimapping between potential peptide origins, whilst missing database entries result in false negatives, and we provide bioinformatics tools to address these issues.

Using Sequoia and SPIsnake we increased the percentage of discoverable peptides in an analyte and improved the separation of target and decoy distributions. This improved the MSFragger search engine performance by filtering out unsupported peptide sequences and yielding more peptides compared to commonly employed post-filtering strategies (15, 51, 52, 53, 94, 131, 132). However, database pre-filtering requires careful calibration to preserve correct peptide sequences and optimize the search space. SPIsnake's RT and HLA-I binding affinity filters are dataset-specific, and the thresholds must balance between retaining true sequences and excluding false ones. Excessive pre-filtering will inevitably remove the correct targets from the search space, which suggests the main utility of pre-filtering in conservative removal of the larger set of most likely incorrect targets, and applying the PSM scoring to disambiguate the remaining better-informed candidates. In the future, enhanced RT and HLA-I binding predictors could further increase database information content.

Additional to database information content, FDR estimation strategies need to be critically assessed in inflated search spaces. The statistical assumptions of typical target-decoy approaches that rely on reversed target databases are often violated, resulting in underestimation of FDRs. Pre-filtering approaches could result in decoy score distributions that are not derived from the same score distribution as the target database. To address the issue of unfair decoys in our proof-of-concept analysis using MSFragger, we applied all SPIsnake pre-filtering steps used upon the target databases to the reversed sequence database as well, ensuring that target and decoy database were equally informed and allowing robust FDR estimation.

*De novo* sequencing and exhaustive methods are often paired with stratified hierarchical approaches to manage large search spaces (12, 20, 27, 45, 50, 128). Several approaches prioritize peptides from smaller, more defined strata (15, 44, 45, 128, 133, 134), but the process is complex and subjective, influenced by pre-existing assumptions about strata priority. Tools like Sequoia and SPIsnake could help informing these hierarchies. Both *de novo* peptide sequencing and an exhaustive approach to extremely large search spaces in noncanonical immunopeptidomics, result in a competition of hundreds to thousands isobaric peptide sequences for a given mass spectrum, many of which can have very similar quality scores. To identify the most likely PSM, hierarchical ranking based on the peptide origins have been utilized (12, 20, 27, 45, 50, 128). In such approaches, sequence candidates that map to canonical CDS main ORF are preferred over sequences that map to, e.g., introns. However, construction and hierarchies of subsequent strata varies across studies and is often driven by assumptions about identification priority, challenging the correct assignment for multimapping peptides and overlapping proteogenomic origins. The assignment hierarchy is often guided by strata sizes, prioritizing the smallest strata during spectral matching over the origins from larger strata (15, 44, 45, 128, 133, 134). Therefore, strata sizes must be carefully evaluated together with the supporting information that was used to generate them, *e.g*., using SPIsnake.

Furthermore, sequence candidates that map to more than one stratum, *i.e.*, multi-mappers explored by SPIsnake, reduce the effective strata sizes depending on the employed hierarchies. Strata sizes and score distributions should be carefully evaluated in order to derive the suitable statistical framework and to appropriately estimate FDRs. The latter are usually estimated using target-decoy approaches. It is unclear, however, how imposed hierarchies in stratified approaches impact the target-decoy approach. In this study, we utilized a combined strategy that prioritizes canonical peptides over noncanonical peptides in a probabilistic manner rather than in a stratified hierarchical approach for sensitive canonical peptide identification, while also exploring noncanonical peptides. Although this combined strategy improved the quality of noncanonical peptide assignments, some low-quality assignments persisted. The investigated approach was limited using a standard search engine scoring approach, aiming to understand the impact of SPIsnake’s pre-filtering approach on search engine performance. By comparing the combined strategy with the most stringent MSFragger group-specific FDR estimation, we observed the loss of all noncanonical PSMs. The sensitivity of spectral rescoring to the lack of “good” targets in the training set is revealed in the group-specific strategy. On the contrary, the combined strategy provided a well-informed positive class for rescoring and could be further expanded to avoid internally heterogenous strata, where the identification of smaller well-informed origins could be compromised by the numerous low-quality targets. In either case, all employed strategies benefited from the SPIsnake pre-filtering in terms of increased peptide recall, while maintaining spectral quality.

We believe that the increased sensitivity and reliability brought by feature-based rescoring approaches (*e.g.,* Percolator (135, 136)), especially when connected with spectral prediction (*e.g.,* inSPIRE, PEPSeek, Oktoberfest, MS2Rescore (96, 97, 102, 120, 121)), could pave the way to further improvements in the identification quality in inflated search spaces. Inclusion of the stratum and ORF properties as features could be a balanced unsupervised solution to avoid hard-coded prioritization and compensate for potential strata heterogeneity whilst avoiding the problem of aggregation of multiple-record strata. In turn, strategies using spectral rescoring could achieve higher performance when using Sequoia and SPIsnake to generate informed search spaces prior to search engine application.

In summary, combining transcriptome-informed databases created by Sequoia with SPIsnake’s dataset-specific pre-filters allows an increase in precision and recall and provides multi-omics support for novel peptide identification. Our open-source pipelines can be used to define and explore the inflated search spaces for a variety of bottom-up proteogenomic applications, while providing full transparency in peptide origin attribution. A clear definition of search space size for canonical and noncanonical, post-translationally modified or unmodified peptide strata in proteomics, and immunopeptidomics can enable the development of appropriate statistical frameworks for FDR computation and aid rescoring approaches to build suitable feature-based models, thus allowing sensitive and high-confident identification of novel peptides and proteoforms in high-throughput proteomics and immunopeptidomics.

## Data Availability

The MS proteomics data have been previously deposited to the ProteomeXchange Consortium *via* the PRIDE (137) partner repository with the dataset identifier PXD031709 (57). The mass spectrometric output files in the original instrument vendor file format have been deposited to the MassIVE partner repository with the dataset identifier MSV000097898.

The RNA-seq data have been deposited in the NCBI Sequence Read Archive database with the accession code PRJNA721129 (57). The Supplemental Tables S1–S20 are available in the Edmond repository (https://doi.org/10.17617/3.2M9RDY).

The algorithm generating all possible *cis-* and homologous *trans-*spliced peptides was originally described by Liepe *et al.* (138) and is implemented in Roetschke *et al.* (19, 20, 23, 25, 26, 27).

Sequoia is available on GitHub under https://github.com/QuantSysBio/sequoia.

SPIsnake is available on GitHub under https://github.com/QuantSysBio/SPIsnake

## Supplemental Data

This article contains supplemental data.

## Conflict of Interests

The authors declare the following financial interests/personal relationships which may be considered as potential competing interests: MM consults for G.S.K. The other authors declare no competing interests.
