# Supplementary material for: An Automated Workflow to Address Proteome Complexity and the Large Search Space Problem in Proteomics and HLA-I Immunopeptidomics
Source: Mol Cell Proteomics. 2025 Jul 21;24(9):101039. doi: 10.1016/j.mcpro.2025.101039 (PMC12397870; doi:10.1016/j.mcpro.2025.101039)
Supplement: Supplementary Material [file mmc1.pdf]

An automated workflow to address proteome complexity and the large search space problem in proteomics and HLA-I immunopeptidomics.

Correspondence to: Juliane Liepe (jliepe@mpinat.mpg.de) & Michele Mishto (michele.mishto@kcl.ac.uk)

Supplementary Table 1	Proteogenomic origins
Supplementary Table 2	Tryptic peptides investigated in the study
Supplementary Table 3	Nonspecific peptides in HLA-I immunopeptidomes investigated in the study
Supplementary Table 4	Comparison of sequence search space of tryptic and nonspecific peptides and the impact of RNA-seq information on search space size
Supplementary Table 5	Novel isoform discovery
Supplementary Table 6	Impact of PTMs on peptide strata size
Supplementary Table 7	Impact of MS1 characteristics on search space for tryptic peptides
Supplementary Table 8	Impact of MS1 characteristics on sequence search space for nonspecific peptides
Supplementary Table 9	Number of spliced peptide sequences per peptide length
Supplementary Table 10	Comparison between theoretical and computationally explored search space
Supplementary Figure 1	Automatic workflow for proteogenomic space definition and filtering
Supplementary Figure 2	Ratios of nonspecific to tryptic peptide search spaces among 8 – 15 residue lone peptides
Supplementary Figure 3	Influence of sequence-maintaining PTMs on the number of peptide forms
Supplementary Figure 4	Impact of MS1 characteristics on sequence search space for nonspecific peptides
Supplementary Figure 5	Predicted HLA-I-peptide binding affinity in the canonical immunopeptidomes of K562-A*02:01 and K562-B*07:02 immunopeptidomes
Supplementary Figure 6	PCPS
Supplementary Figure 7	Comparison of analytically and empirically explored strata sizes
Supplementary Figure 8	Database size ratios and score distributions of target and decoy PSM scores for naïve method
Supplementary Figure 9	Strategies for noncanonical peptide identification
Supplementary Figure 10	Strategies for noncanonical peptide identification applied to the B721.221 cell line
Supplementary Figure 11	Additional benchmarks for noncanonical peptide identification strategies
Supplementary Figure 12	Comparisons of noncanonical PSM quality for different FDR estimation methods

SOURCE DATA

Source Data Tables 1-20 are available in the Edmond repository: https://doi.org/10.17617/3.2M9RDY

Source Data Table 1| Sequoia constructed reference databases. This file contains GENCODE driven and RNA-seq informed reference databases in FASTA format and ORF annotations in tabular format (37.8 GB on disk). RNA-seq data were derived from K562 cell line and used for reference-guided transcriptome assembly, quantification and ORF prediction. Provided are the GENCODE v33 reference ORF translations, the expressed sub-set of reference proteome in RNA-seq and translation sequences of predicted coding ORFs from novel isoforms. Also included are the exhaustively searched intergenic and transcriptomic ORF translation sequences on expressed and full reference transcript sets.

Source Data Table 2| SPIsnake user parameter settings. This file contains a list of folders with pre-set SPIsnake job inputs, with all corresponding settings and hyperparameters used in this study.

Source Data Table 3| Ratios of nonspecific/tryptic peptides in K562 cell line. An .xlsx table, reporting the numbers of unique peptides in GENCODE-informed and RNA-seq informed strata, across filtering steps. Includes the ratios of nonspecific/tryptic peptide counts.

Source Data Table 4| Gene fusion detection in K562 cell line. An .xlsx table, derived from the Arriba gene fusion detection tool, reporting the identified gene fusions and corresponding biological replicates of K562 cells.

Source Data Table 5| 703 PTMs investigated in Fig. 2d and Supplementary Fig. 3e. PTMs from Unimod database in tabular format.

Source Data Table 6l PTM-peptide forms per protein. Number of unmodified peptides and PTM combinations in unfiltered search spaces and upon molecular weight filter.

Source Data Table 7l Strata search space sizes across SPIsnake filtering steps in K562. Number of unique peptides derived by tryptic or proteasomal digestion from RNA-seq informed and uninformed search spaces for all peptide lengths. Strata reduction during data-driven filtering is reported for K562-A*02:01 and K562-B*07:02 cell lines.

Source Data Table 8l IC50 cutoffs. Reported are the numbers of peptides identified in PEAKS search of K562 HLA-I immunopeptidome at 1% FDR, upon 5000 nM cut-off. The absolute and relative quantities are reported per haplotype and peptide length and as a summary.

Source Data Table 9l Number of unique *cis*-spliced and non-spliced peptides per protein length, peptide length and maximum intervening sequence length. The figures refer to peptides either unfiltered, filtered based on molecular weight (MW filtered), additionally filtered by retention time prediction (MW-RT-filtered), or additionally filtered by HLA-I-peptide binding affinity predictions (MW-RT-HLA-I binding filtered). This file refers to **Fig. 4a**.

Source Data Table 10l Peptide multi-mapping within strata. This file contains the numbers of peptides per number of multimapping origins within proteogenomic strata. For exhaustive transcriptomic and genomic ORFs the peptide mapping was checked on ORF level, for reference proteome, CDS off-frame and cis25-spliced peptides the mapping was checked also on gene level. Both unfiltered peptide mapping events are counted, as well as the numbers of peptides after SPIsnake MW-RT-HLA-I binding filtering per replicate. Data refers to K562-A*02:01 and K562-B*07:02 cell lines.

Source Data Table 11l Comparison between theoretical and computationally explored strata sizes. Number of unique unspecific peptide sequences and number of theoretical peptide sequences for RNA-seq informed and GENCODE driven strata is shown per peptide length. This table refers to **Supplementary Fig. 7e**. The data refers to K562 cell line.

Source Data Table 12l Peptide multi-mapping across strata pairs. This file contains the pairwise comparisons of distinct peptide sets between proteogenomic strata. For every pair of databases, peptide length and catalytic rule, provided are the numbers of peptides distinct for each stratum as well as the intersection size and ratios of intersection to each set. Unfiltered estimates are reported together with SPIsnake-filtered sets per replicate. Data refers to K562-A*02:01 and K562-B*07:02 cell lines.

Source Data Table 13l Strata search space sizes across SPIsnake filtering steps in B721.221. Number of unique peptides derived by tryptic or proteasomal digestion from RNA-seq informed and uninformed search spaces for all peptide lengths. Strata reduction during data-driven filtering is reported for B721.221 cell line expressing HLA-A02:01 or HLA-B07:02.

Source Data Table 14l FASTA Files of MW-filtered canonical and expanded reference databases. The zip file contains two FASTA files of target and decoy peptide sequences for canonical and expanded databases. Decoy sequences are indicated by the label "rev_" in the FASTA header.

Source Data Table 15l FASTA Files of MW-RT-filtered canonical and expanded reference databases. The zip file contains two FASTA files of target and decoy peptide sequences for canonical and expanded databases. Decoy sequences are indicated by the label "rev_" in the FASTA header.

Source Data Table 16l FASTA Files of MW-RT-affinity-filtered canonical and expanded reference databases. The zip file contains two FASTA files of target and decoy peptide sequences for canonical and expanded databases. Decoy sequences are indicated by the label "rev_" in the FASTA header.

Source Data Table 17l MSFragger original search engine outputs. The zip file contains subfolders each containing 'pepXML' output files from MSFragger for the MS raw files which were searched. Folder titles indicate the model system, whether the search results come from the canonical or expanded reference database search and which SPIsnake filters were applied.

Source Data Table 18l Peptide sequences identified with the naïve strategy. This file contains .csv files of peptide sequences filtered for 1% FDR identified with the naïve strategy canonical and expanded reference databases using MW-filters, MW-RT-filters or MW-RT-HLA-I binding filters. Furthermore, peptide sequences identified with MSFragger at 1% FDR and subsequently filtered for RT and HLA-I-peptide binding affinity (post-filter strategy) are provided. All PSMs for a single peptide are reported with

the source file, scan number, peptide sequence, retention time, and q-value reported. Folder titles indicate if the search results come from the canonical or expanded reference database search and which SPIsnake filters and/or post-filters were applied.

Source Data Table 19l Peptide sequences identified with the combined strategy. This file contains .csv files of peptide sequences filtered for 1% FDR identified with the combined strategy using either MW filtered, MW-RT filtered or MW-RT-HLA-I binding filtered reference databases, respectively. Furthermore, peptide sequences identified with MSFragger at 1%FDR and subsequently filtered for RT and HLA-I-peptide binding affinity (post-filter strategy) are provided. All PSMs for a single peptide are reported with the source file, scan number, peptide sequence, RT, and q-value reported. Folder titles indicate if the search results come from the canonical or expanded reference database search and which SPIsnake filters and/or post-filters were applied.

Source Data Table 20l Peptide sequences identified with the group specific FDR estimation. This file contains .csv files of peptide sequences filtered for 1% FDR identified with the group specific FDR estimation combined strategy using either MW filtered, MW-RT filtered or MW-RT-HLA-I binding filtered reference databases, respectively. Furthermore, peptide sequences identified with MSFragger at 1%FDR and subsequently filtered for RT and HLA-I-peptide binding affinity (post-filter strategy) are provided. All PSMs for a single peptide are reported with the source file, scan number, peptide sequence, RT, and q-value reported. Folder titles indicate if the search results come from the canonical or expanded reference database search and which SPIsnake filters and/or post-filters were applied.

SUPPLEMENTARY TABLES

**Protogenomic origin**	**Description**
CDS main ORF	mRNA main-frame translation (canonical reference proteome)
(CDS) off-frame	mRNA off-frame translation
lncRNA	Non-coding RNA translation
5'-UTR	ORFs in 5' untranslated regions
3'-UTR	ORFs in 3' untranslated regions
intronic	Nested within introns or due to intron retention in mRNA
intergenic	Unannotated ORFs between known genes
de novo isoforms	Predicted ORFs located on novel isoforms assembled from RNA sequencing data using the Sequoia pipeline

Supplementary Table 1I Proteogenomic origins. Description of origins defined as strata and investigated in this study.

Proteogenomic origin	PTM	RNA-seq informed	MW filtered	MW-RT filtered
CDS main frame	n = 0	Y / N	Y	Y
	n = 8	Y / N	Y	N
	n = 703	Y / N	Y	N
CDS off-frame	n = 0	Y / N	Y	Y
	n = 8	Y / N	Y	N
lncRNA	n = 0	Y / N	Y	Y
	n = 8	Y / N	Y	N
5’-UTR	n = 0	Y / N	Y	Y
	n = 8	Y / N	Y	N
3’-UTR	n = 0	Y / N	Y	Y
	n = 8	Y / N	Y	N
intronic	n = 0	Y / N	Y	Y
	n = 8	Y / N	Y	N
intergenic	n = 0	N	Y	Y
	n = 8	N	Y	N
de novo isoforms	n = 0	Y	Y	Y
	n = 8	Y	Y	N

Supplementary Table 2l Tryptic peptides investigated in the study. The tryptic search spaces were filtered either by expression in RNA-seq and further MW filtered or MW-RT filtered, respectively, as indicated by binary labels (“Y” – yes; “N” – no). PTM expanded search spaces did not undergo filtering by RT. RT – retention time; MW – molecular weight.

Proteogenomic origin	PTMs	RNA-seq informed	MW filtered	MW-RT filtered	MW-RT-HLA-I binding filtered
CDS main frame	n = 0	Y / N	Y	Y	Y
	n = 8	Y / N	Y	N	N
	n = 703	Y / N	Y	N	N
	cis25-spliced	Y	Y	Y	Y
	cis50-spliced	Y	Y	Y	Y
	cis100-spliced	Y	Y	Y	Y
	cis200-spliced	Y	Y	Y	Y
CDS off-frame	n = 0	Y / N	Y	Y	Y
	n = 8	Y / N	Y	N	N
lncRNA	n = 0	Y / N	Y	Y	Y
	n = 8	Y / N	Y	N	N
5'-UTR	n = 0	Y / N	Y	Y	Y
	n = 8	Y / N	Y	N	N
3'-UTR	n = 0	Y / N	Y	Y	Y
	n = 8	Y / N	Y	N	N
intronic	n = 0	Y / N	Y	Y	Y
	n = 8	Y / N	Y	N	N
intergenic	n = 0	N	Y	Y	Y
	n = 8	N	Y	N	N
de novo isoforms	n = 0	Y	Y	Y	Y
	n = 8	Y	Y	N	N

Supplementary Table 3l Nonspecific peptides in HLA-I immunopeptidomes investigated in the study. The nonspecific search spaces were filtered either by expression in RNA-seq and further MW filtered, MW-RT filtered or MW-RT-HLA-I binding filtered, respectively, as indicated by binary labels ("Y" – yes; "N" – no). PTM expanded search spaces did not undergo filtering by retention time. RT – retention time; MW – molecular weight.

stratum	number of unique peptide sequences			
	**tryptic GENCODE driven**	**tryptic RNA-seq informed**	**nonspecific GENCODE driven**	**nonspecific RNA-seq informed**
CDS main ORF	2.82E+06	1.84E+06	9.17E+07	5.95E+07
intergenic	3.53E+08	N.A.	7.82E+09	N.A.
intronic	1.63E+08	5.93E+07	3.93E+09	1.43E+09
lncRNA	5.18E+07	8.41E+06	1.20E+09	1.92E+08
CDS frameshift	7.42E+06	4.72E+06	3.07E+08	1.98E+08
5'UTR	8.33E+05	4.28E+05	1.66E+07	8.39E+06
3'UTR	4.69E+06	2.94E+06	1.10E+08	6.92E+07
de novo isoforms	N.A.	5.54E+05	N.A.	1.82E+07

Supplementary Table 4l Comparison of sequence search space of tryptic and nonspecific peptides and the impact of RNA-seq information on search space size. Tryptic peptides computed were 5-30 residue long, covering the typical length range observed in tryptic proteome digests. Nonspecific peptides were 8-15 residue long, covering the typical length range observed in HLA-I immunopeptidomes. This table refers to **Fig. 2b-c**.

genes	transcripts	ORFs	gffcompare class code	description
1	1	1	c	contained in reference (intron compatible)
773	875	471	k	containment of reference (reverse containment)
271	293	268	m	retained intron(s), all introns matched or retained
448	629	541	n	retained intron(s), not all introns matched/covered
3405	5790	4975	j	multi-exon with at least one junction match
50	56	21	o	other same strand overlap with reference exons
102	128	22	x	exon overlap on the opposite strand (like o or e but on the opposite strand)
307	320	46	i	fully contained within a reference intron
11	14	4	y	contains a reference within its introns
49	49	12	p	possible polymerase run-on (no actual overlap)
449	504	86	u	none of the above (unknown, intergenic)

Supplementary Table 5I Novel isoform discovery. Shown are the numbers of genomic loci, transcripts and predicted ORFs identified through the novel isoform assembly from RNA-seq data. GENCODE reference v33 was used for genomic alignment, isoform assembly and reference matching. All transcripts that did not match reference completely, *i.e.,* by exact match of intron chain (gffcompare class code “=”) were searched for coding ORFs. The numbers are reported per gffcompare class code, that defines the type of transcriptomic overlap of novel isoforms as compared to reference transcripts.

status	stratum	number of unique sequences					
		tryptic	tryptic with 8 PTMs	nonspecific	nonspecific with 8 PTMs	nonspecific with 703 PTMs	cis25-spliced
GENCODE driven	CDS main ORF	2.82E+06	5.79E+07	9.17E+07	1.28E+09	2.99E+13	N.A.
	intergenic	3.53E+08	1.56E+10	7.82E+09	1.12E+11	N.A.	N.A.
	intronic	1.63E+08	3.53E+09	3.93E+09	5.51E+10	N.A.	N.A.
	lncRNA	5.18E+07	1.11E+09	1.20E+09	1.74E+10	N.A.	N.A.
	CDS frameshift	7.42E+06	1.40E+08	3.07E+08	4.42E+09	N.A.	N.A.
	3'UTR	4.69E+06	9.18E+07	1.10E+08	1.55E+09	N.A.	N.A.
	5'UTR	8.33E+05	1.17E+07	1.66E+07	1.65E+08	N.A.	N.A.
RNA-seq informed	*de novo* isoforms	5.54E+05	1.07E+07	1.82E+07	2.37E+08	N.A.	N.A.
	CDS main ORF	1.84E+06	3.64E+07	5.95E+07	7.97E+08	1.94E+13	2.43E+10
	intronic	5.93E+07	1.23E+09	1.43E+09	1.92E+10	N.A.	N.A.
	lncRNA	8.41E+06	1.72E+08	1.92E+08	2.65E+09	N.A.	N.A.
	CDS frameshift	4.72E+06	8.79E+07	1.98E+08	2.57E+09	N.A.	N.A.
	3'UTR	2.94E+06	5.68E+07	6.92E+07	9.73E+08	N.A.	N.A.
	5'UTR	4.28E+05	5.56E+06	8.39E+06	7.87E+07	N.A.	N.A.

Supplementary Table 6l Impact of PTMs on peptide strata size. Shown is the unique peptide search space of unmodified or PTM-modified nonspecific peptides. PTMs explored were either M-oxidation, N/Q deamidation, peptide N-terminal acetylation, carbamidomethylation of cysteine (C), and S, T, Y-phosphorylation (8 PTMs), a set of 703 PTMs, and PCPS. The latter was restricted to intervening sequence length of maximum 25 amino acids (cis25-spliced). Tryptic peptides computed were 5-30 amino acids long, covering the typical length range observed in tryptic proteome digests. Nonspecific peptides were 8-15 amino acids long, covering the typical length range observed in HLA-I immunopeptidomes. This table refers to **Fig. 2d**.

status	stratum	number unique sequences		
		unfiltered	MW filtered	MW-RT filtered
RNA-seq uninformed	CDS main ORF	2.82E+06	1.59E+06	9.22E+05
	intergenic	3.53E+08	2.10E+08	1.19E+08
	intronic	1.63E+08	9.61E+07	5.59E+07
	lncRNA	5.18E+07	3.04E+07	1.77E+07
	CDS frameshift	7.42E+06	4.46E+06	2.38E+06
	3'UTR	4.69E+06	2.68E+06	1.57E+06
	5'UTR	8.33E+05	4.94E+05	2.76E+05
RNA-seq informed	CDS main ORF	1.84E+06	1.04E+06	6.07E+05
	intronic	5.93E+07	3.45E+07	2.01E+07
	lncRNA	8.41E+06	4.85E+06	2.81E+06
	CDS frameshift	4.72E+06	2.82E+06	1.50E+06
	3'UTR	2.94E+06	1.66E+06	9.77E+05
	5'UTR	4.28E+05	2.54E+05	1.40E+05
	de novo isoforms	5.54E+05	3.15E+05	1.76E+05

Supplementary Table 7l Impact of MS1 characteristics on search space for tryptic peptides. Shown are the numbers of unique peptides per stratum, which are either unfiltered, filtered based on molecular weight (MW filtered), additionally filtered by retention time prediction (MW-RT filtered). This table refers to **Fig. 3a**. Data refers to tryptic proteomics of K562 cell line.

RNA-seq status	PTM status	stratum	number of unique sequences			
			unfiltered	MW filtered	MW-RT filtered	MW-RT-HLA-I binding filtered
RNA-seq uninformed	No PTMs	CDS main ORF	9.17E+07	2.33E+07	7.52E+06	3.80E+05
		intergenic	7.82E+09	1.92E+09	5.07E+08	2.79E+07
		intronic	3.93E+09	1.01E+09	2.67E+08	1.52E+07
		lncRNA	1.20E+09	3.12E+08	8.25E+07	4.49E+06
		CDS frameshift	3.07E+08	8.54E+07	2.42E+07	1.82E+06
		3'UTR	1.10E+08	3.01E+07	8.32E+06	5.55E+05
		5'UTR	1.66E+07	5.07E+06	1.46E+06	1.32E+05
RNA-seq informed	No PTMs	CDS main ORF	5.95E+07	1.52E+07	4.99E+06	2.52E+05
		intronic	1.43E+09	3.74E+08	9.95E+07	5.77E+06
		lncRNA	1.92E+08	5.06E+07	1.36E+07	7.58E+05
		CDS frameshift	1.98E+08	5.49E+07	1.56E+07	1.16E+06
		3'UTR	6.92E+07	1.90E+07	5.25E+06	3.49E+05
		5'UTR	8.39E+06	2.60E+06	7.59E+05	7.06E+04
		de novo isoforms	1.82E+07	4.68E+06	1.55E+06	7.79E+04
	8 PTMs	CDS main ORF	7.97E+08	4.38E+07	0.00E+00	0.00E+00
		intronic	1.92E+10	1.06E+09	0.00E+00	0.00E+00
		lncRNA	2.65E+09	1.50E+08	0.00E+00	0.00E+00
		CDS frameshift	2.57E+09	1.52E+08	0.00E+00	0.00E+00
		3'UTR	9.73E+08	5.40E+07	0.00E+00	0.00E+00
		5'UTR	7.87E+07	6.16E+06	0.00E+00	0.00E+00
		de novo isoforms	2.37E+08	1.33E+07	0.00E+00	0.00E+00
	cis25-spliced	CDS main ORF	2.43E+10	4.87E+09	1.51E+09	7.23E+07

Supplementary Table 8l Impact of MS1 characteristics on sequence search space for nonspecific peptides. Shown are number of unique peptides per stratum, which are either unfiltered, filtered based on molecular weight (MW filtered), additionally filtered by retention time prediction (MW-RT filtered), or additionally filtered by HLA-I binding predictions (MW-RT-HLA-I binding filtered). Analysis was performed on GENCODE driven strata and on RNA-seq informed strata, respectively. Data refers to HLA-I immunopeptidomics of K562-B*07:02 cell lines.

peptide length (num. aa)	maximum 20 amino acid combinations	all *trans*-splicing events	expected unique *trans*-spliced	all *cis*-splicing events	expected unique *cis*-spliced	all cis25-splicing events	unique cis25-spliced in proteome
8	1.15E+18	1.04E+15	2.56E+10	1.08E+11	2.52E+10	5.91E+09	1.56E+09
9	1.22E+19	1.19E+15	5.12E+11	1.23E+11	1.09E+11	6.73E+09	2.14E+09
10	1.00E+20	1.34E+15	1.02E+13	1.38E+11	1.37E+11	7.55E+09	2.54E+09
11	6.73E+20	1.48E+15	2.05E+14	1.53E+11	1.53E+11	8.37E+09	2.90E+09
12	3.83E+21	1.62E+15	1.34E+15	1.68E+11	1.68E+11	9.18E+09	3.25E+09
13	1.90E+22	1.77E+15	1.75E+15	1.82E+11	1.82E+11	9.99E+09	3.61E+09
14	8.37E+22	1.91E+15	1.91E+15	1.97E+11	1.97E+11	1.08E+10	3.96E+09
15	3.33E+23	2.05E+15	2.05E+15	2.12E+11	2.12E+11	1.16E+10	4.32E+09

Supplementary Table 9| Number of spliced peptide sequences per peptide length. We here compare the number of spliced peptide sequences per peptide length considering cis-spliced peptides with a maximum intervening sequence length of 25 amino acids (cis25-spliced), all *cis*-spliced peptides and all *trans*-spliced peptides for CDS main ORF. All *cis*- and all *trans*-spliced peptides were theoretically determined (see Methods) and hence present an upper bound, while cis25-spliced were determined based on actual unique sequences. This table refers to **Fig. 4b-c**.

stratum	enzyme type	RNA-seq status	theoretical	unique	unique / theoretical, %
CDS main ORF	cis25-spliced	RNA-seq informed	7.01E+10	2.43E+10	34.6%
CDS main ORF	nonspecific		1.36E+08	5.95E+07	43.9%
CDS frameshift			6.13E+08	1.98E+08	32.2%
5’UTR			9.41E+06	8.39E+06	89.2%
intronic			1.80E+09	1.43E+09	79.6%
lncRNA			2.27E+08	1.92E+08	84.9%
3’UTR			7.66E+07	6.92E+07	90.3%
de novo isoforms			3.10E+07	1.82E+07	58.6%
CDS main ORF		GENCODE driven	2.55E+08	9.17E+07	36 %
CDS frameshift			1.29E+09	3.07E+08	23.8%
5’UTR			1.89E+07	1.66E+07	87.8%
3’UTR			1.22E+08	1.10E+08	89.7%
intronic			4.95E+09	3.93E+09	79.3%
lncRNA			1.45E+09	1.20E+09	83 %
intergenic			1.14E+10	8.19E+09	71.6%

Supplementary Table 10l Comparison between theoretical and computationally explored search space. Number of unique nonspecific peptide sequences and number of theoretical peptide sequences for RNA-seq informed and GENCODE driven peptide strata are shown. This table refers to **Supplementary Fig. 7d**.

Extended Data Figures

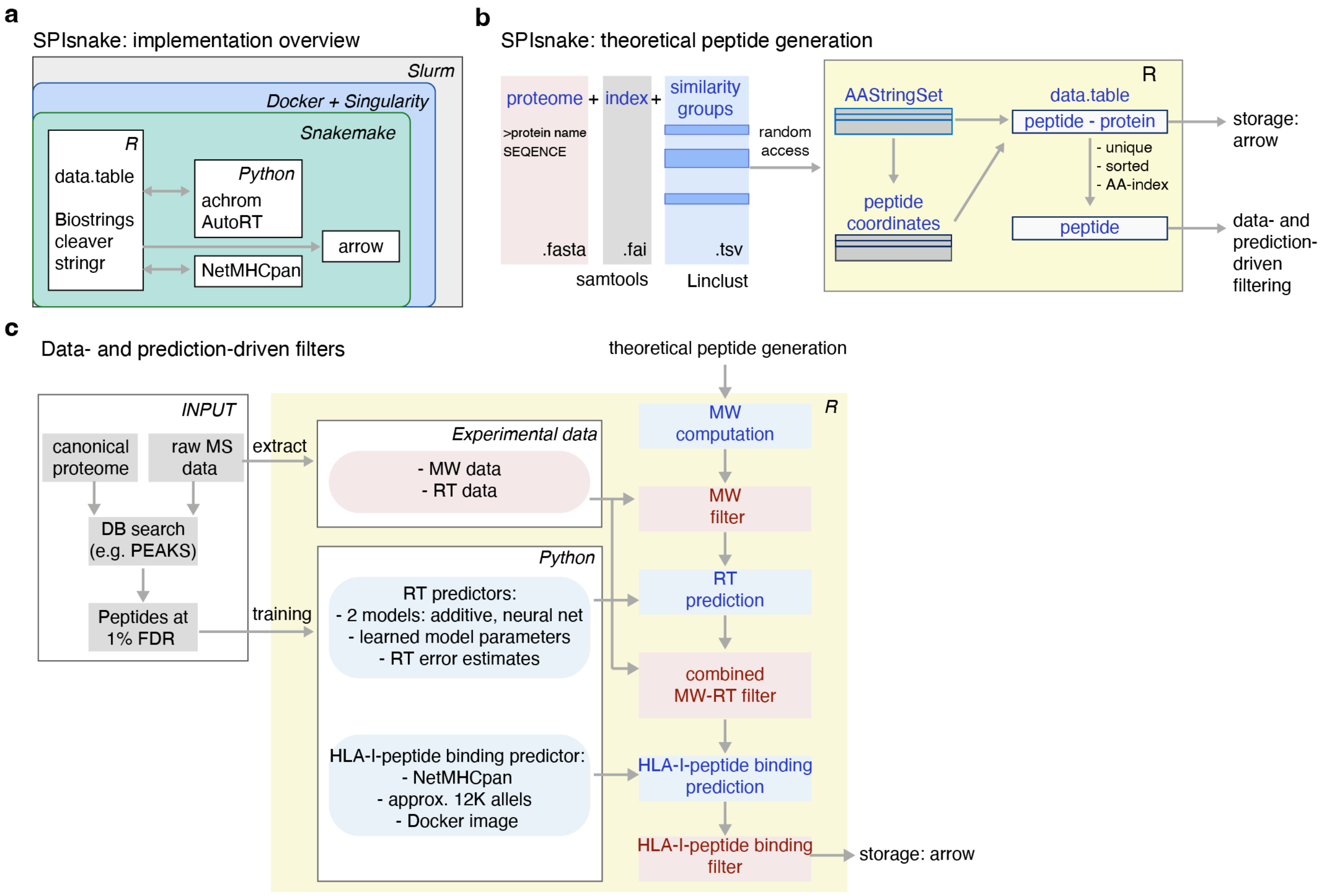

Supplementary Figure 1l Automatic workflow for proteogenomic space definition and filtering. (**a**) SPIsnake implementation overview. A Snakemake workflow is used to call R/Python scripts and standalone software with its dependencies installed into a Docker image. R is the main language of the pipeline, that works with FASTA files, performs string operations, data-driven filtering and communicates with standalone RT and HLA-I-peptide affinity predictors. Larger-than-memory operations use arrow and duckdb backends. Cluster execution is facilitated with Slurm workload manager. (**b**) SPIsnake peptide generation schematic. Proteomes are clustered to identify the groups of similar proteins and indexed to enable random reading access. Protein sequences are selectively read and undergo an *in silico* enzymatic digestion. For PCPS, the positions of splice-reactant coordinates are generated on-the-fly. Peptide-protein mappings are saved as arrow datasets in columnar parquet format. Next, a unique set of peptides undergoes further data-driven filtering. (**c**) SPIsnake data- and prediction-driven filtering schematic. A conventional MS search with a reference proteome at 1% FDR is done to match the RTs to peptide sequences. These are used to train a RT prediction model (additive model or a neural network) and the prediction error is used to define the tolerance range for downstream data-driven filtering. MWs of all detected spectra are used to pre-filter the unmodified peptide sequences from the *in silico* enzymatic digestion and their variable PTM combinations. All other unmodified peptides undergo filtering such that given both the RT model prediction error range and MS-I precursor mass tolerance, they could explain the observed MS-I data. For HLA-I immunopeptidomes, NetMHCpan can predict the binding affinity further filter the peptides given the predicted IC_{50} thresholds.

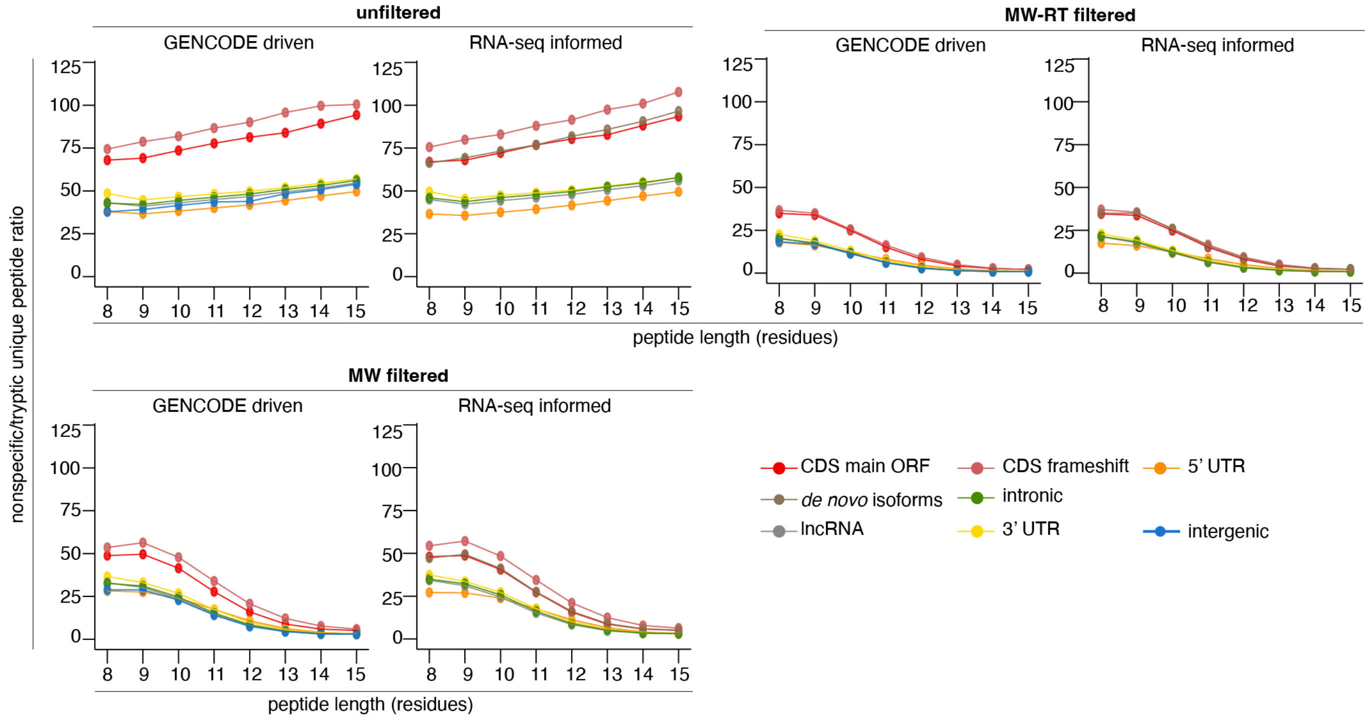

Supplementary Data Figure 2l Ratios of nonspecific to tryptic peptide search spaces among 8 – 15 residue long peptides. Shown are the ratios of nonspecific / tryptic unique peptide counts across proteogenomic origins in the 8-15 residue range, which are either unfiltered, filtered based on molecular weight (MW filtered), or filtered by molecular weight and retention time (MW-RT filtered). The reported numbers are average between the replicates of K562-A*02:01 and K562-B*07:02 immunopeptidomes compared to the number of tryptic peptides from the K562 proteomic dataset. RNA-seq data from K562-A*02:01 and K562-B*07:02 cell lines are used for RNA-informed search spaces.

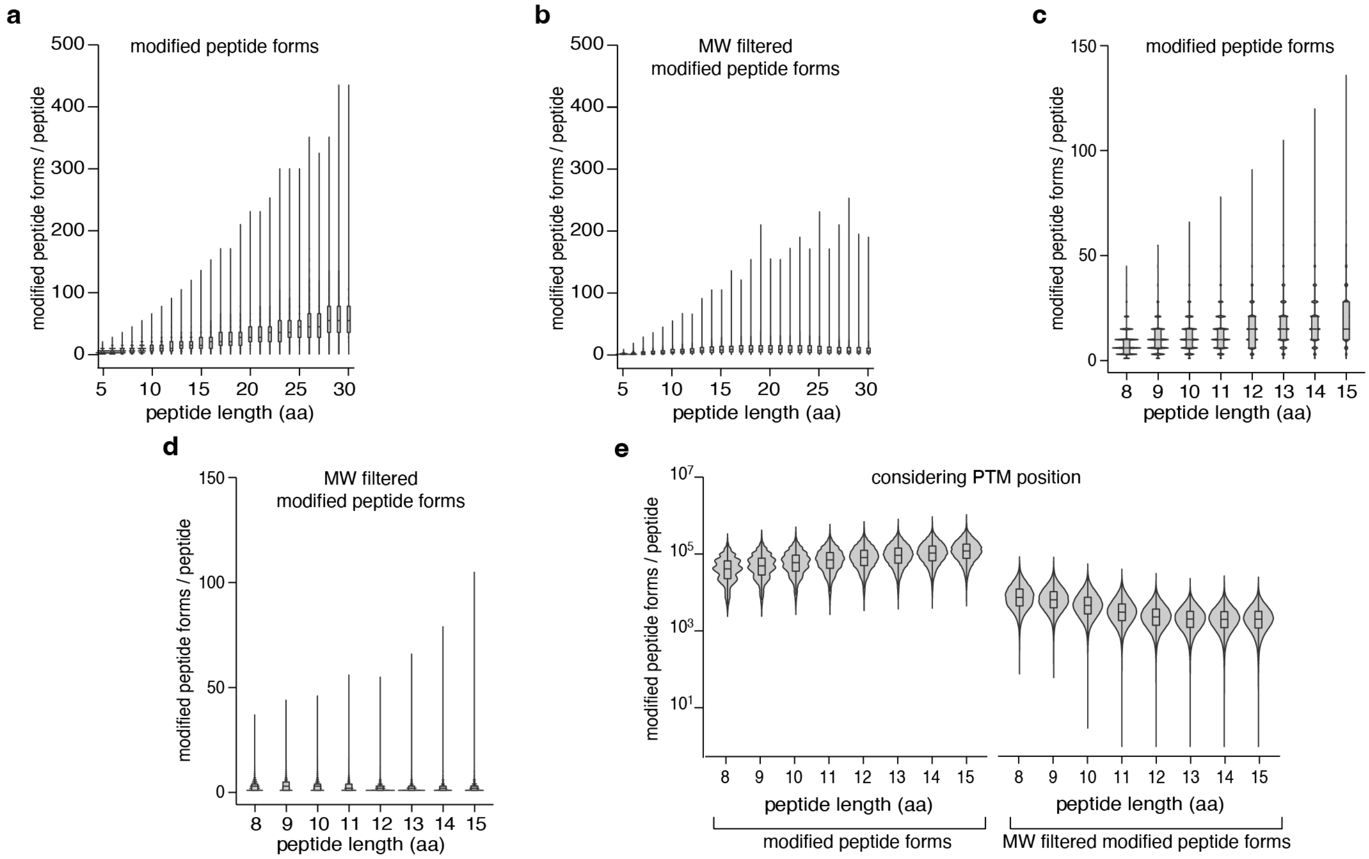

Supplementary Data Figure 3l Influence of sequence-maintaining PTMs on the number of peptide forms. (**a,b**) Number of tryptic unfiltered (**a**) and MW filtered (**b**) peptide forms resulting from the 8 common sequence-maintaining PTMs (*i.e.*, M-oxidation, N/Q deamidation, S/T/Y phosphorylation, N-terminal acetylation and C-carbamidomethylation) considering PTM localization from a given unmodified peptide sequence derived from CDS main ORF. (**c,d**) Number of nonspecific unfiltered (**c**) and MW filtered (**d**) peptide forms resulting from the 8 common sequence-maintaining PTMs considering PTM localization from a given unmodified peptide sequence. (**e**) Number of nonspecific peptide forms resulting from 703 PTMs considering PTM localization from a given unmodified peptide sequence. In (**a-e**) maximum two modifications per sequence are allowed.

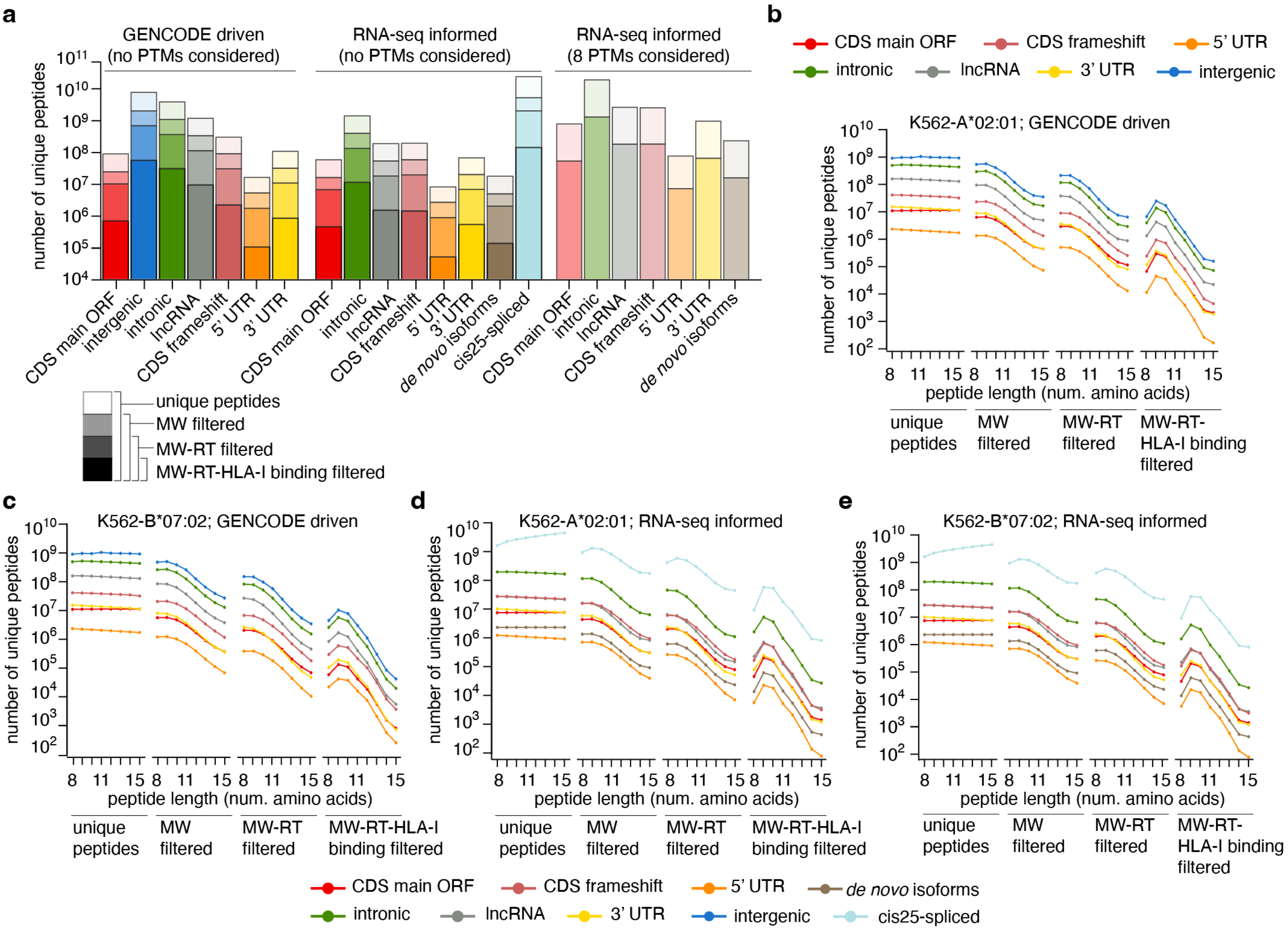

Supplementary Data Figure 4l Impact of MS1 characteristics on sequence search space for nonspecific peptides. (**a-e**) Number of unique peptides per stratum (**a**), or per stratum and peptide length (**b**-**e**), which is either unfiltered (unique peptides), filtered based on molecular weight (MW filtered), filtered by molecular weight and retention time (MW-RT filtered), or additionally filtered by HLA-I-peptide binding affinity predictions (MW-RT-HLA-I binding filtered) is shown. Analysis represents either mean over 3 biological replicates, measured in 2 technical replicates derived from K562-A*02:01 HLA-I immunopeptidome (**a,b,d**), or mean over 2 biological replicates, measured in 1 and 2 technical replicates, respectively, derived from K562-B*07:02 HLA-I immunopeptidome (**c,e**). Analysis is performed on strata derived from full GENCODE driven (**a-c**), or RNA-seq informed peptide strata (**a,d,e**).

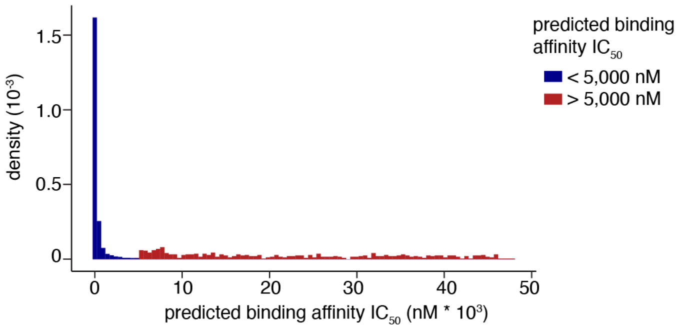

Supplementary Data Figure 5I Predicted HLA-I-peptide binding affinity in the canonical immunopeptidomes of K562-A*02:01 and K562-B*07:02 immunopeptidomes. Shown is the distribution of HLA-I peptide affinities, predicted with NetMHCpan4.1. The peptides were identified from K562-A*02:01 and K562-B*07:02 immunopeptidomes using PEAKS DB at 1% FDR, and a non-binder cutoff of 5,000 nM was color-coded.

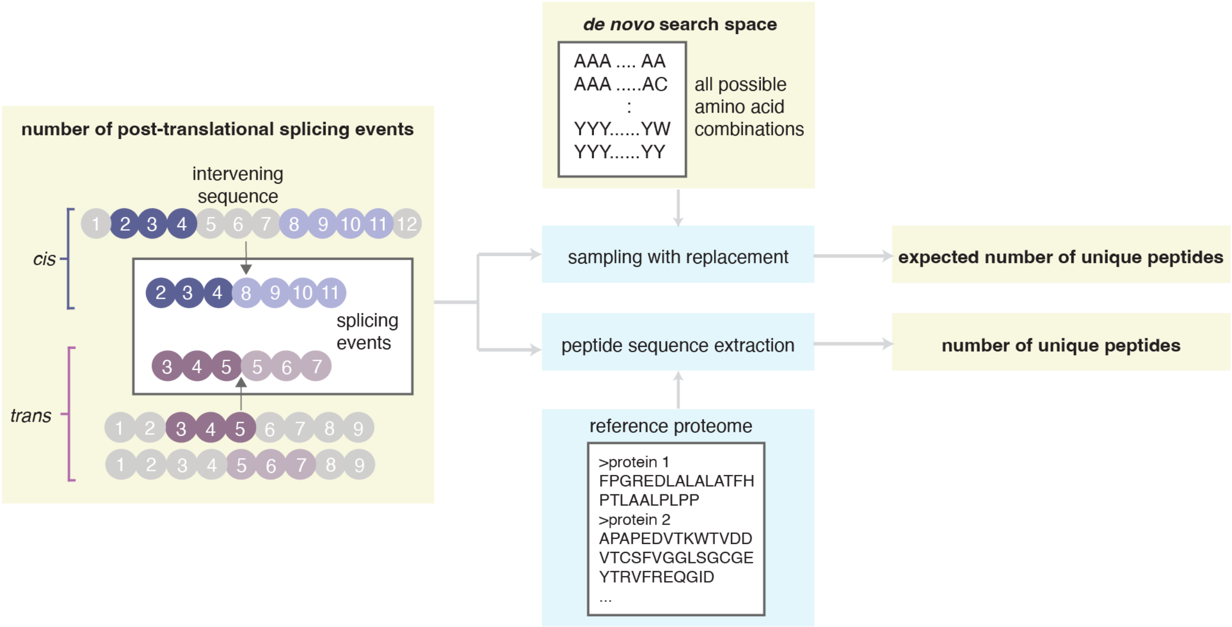

Supplementary Data Figure 6I PCPS. Illustration of relationship between *de novo* search space, number of post-translational splicing events, number of unique post-translationally spliced peptides and expected number of unique post-translationally spliced peptides.

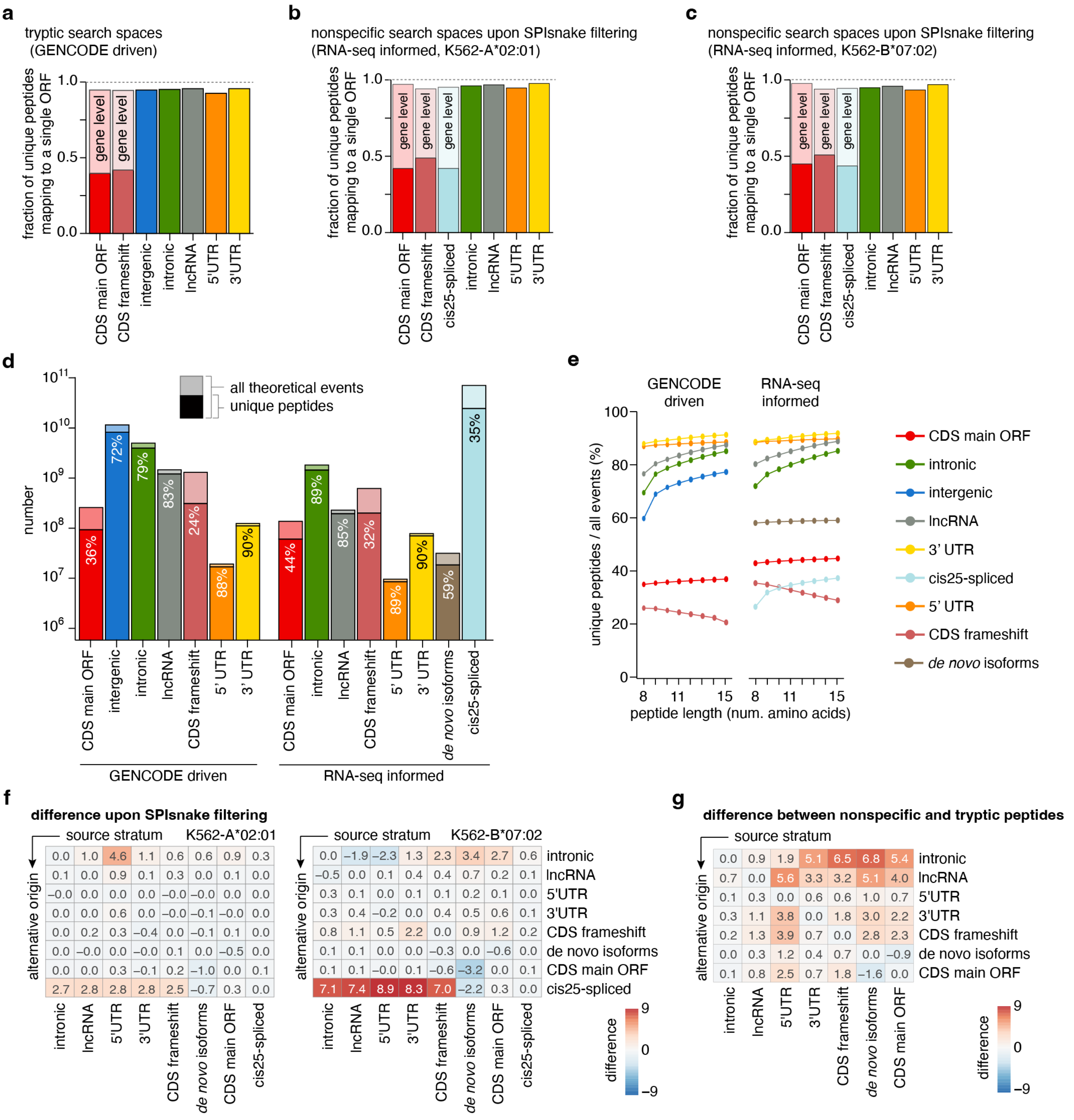

Supplementary Data Figure 7I Comparison of analytically and empirically explored strata sizes. (**a**) Multimapping within strata for tryptic sequences derived from GENCODE. (**b-c**) Multimapping within strata for nonspecific sequences derived from RNA-seq informed strata. Nonspecific peptides have been filtered by MW, RT and HLA-I-peptide binding affinity prediction for K562-A*02:01 (**b**) and K562-B*07:02 (**c**) derived immunopeptidomes. (**d**) Relative reduction of number of all peptide hydrolysis and splicing events due to sequence identity for RNA-seq informed and GENCODE driven strata is displayed. Percentages indicate the number of unique nonspecific peptide sequences (empirically determined) relative to the number of all possible hydrolysis or splicing events (analytically determined). Analysis was carried out on unfiltered strata, without PTMs, but including cis25-spliced peptides derived from RNA-seq informed CDS main ORF (**e**) Search space reduction due to sequence identity, calculated as ratio of unique peptide sequences to the number of possible hydrolysis/splicing events for same strata as in (**d**). Heatmaps in (**f**) indicate the absolute difference in the fraction of peptides that multimap to an alternative stratum comparing SPIsnake MW-RT-HLA-I binding filtered strata against unfiltered strata. K562-A*02:01 and K562-B*07:02 cell lines derived immunopeptidomes were used for SPIsnake filtering. Heatmap in (**g**) indicate the absolute difference in the fraction of peptides that multimap to an alternative stratum comparing tryptic peptides against nonspecific peptides.

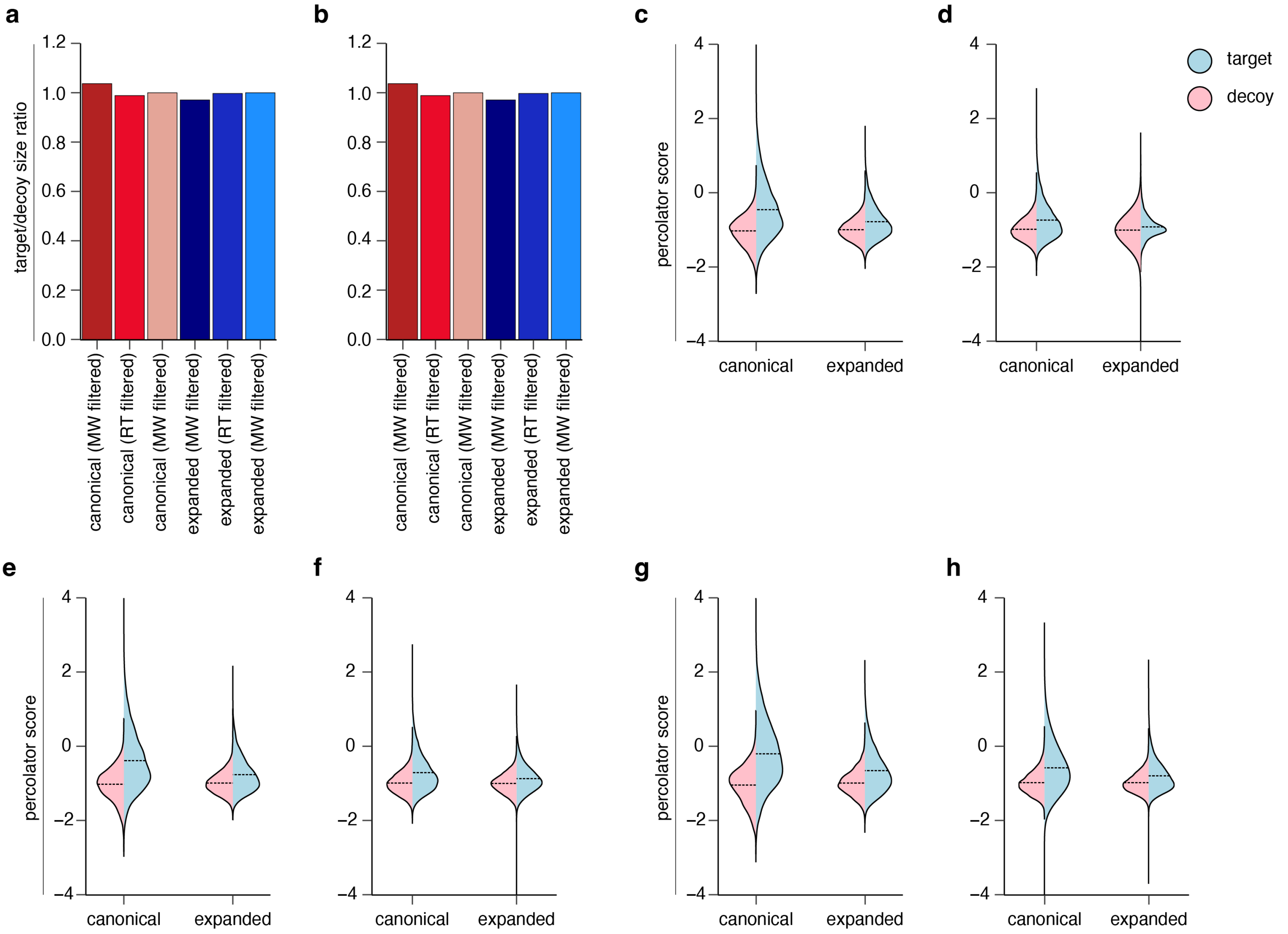

Supplementary Data Figure 8l Database size ratios and score distributions of target and decoy PSM scores for naïve method. (**a-b**) The number of unique peptides present in the target dataset divided by the number of unique peptides in the decoy database for canonical and expanded databases with various filtering strategies for the immunopeptidomes of K562-B*07-02 (**a**) and B721.221-B*07-02 (**b**). (**c**-**h**) Distribution of Percolator scores for target and decoy PSMs after MSFragger search and Percolator rescoring of MS data from the K562-B*07-02 (**c, e, g**) and B721.221-B*-07:02 (**d, f, h**) cell line immunopeptidomes, when confronted with a canonical reference database (RNA-informed CDS main ORF) or an expanded reference database (multiple RNA-seq informed strata together) with MW (**c, d**), RT (**e, f**), and HLA-I-peptide binding affinity (**g, h**) filtering applied.

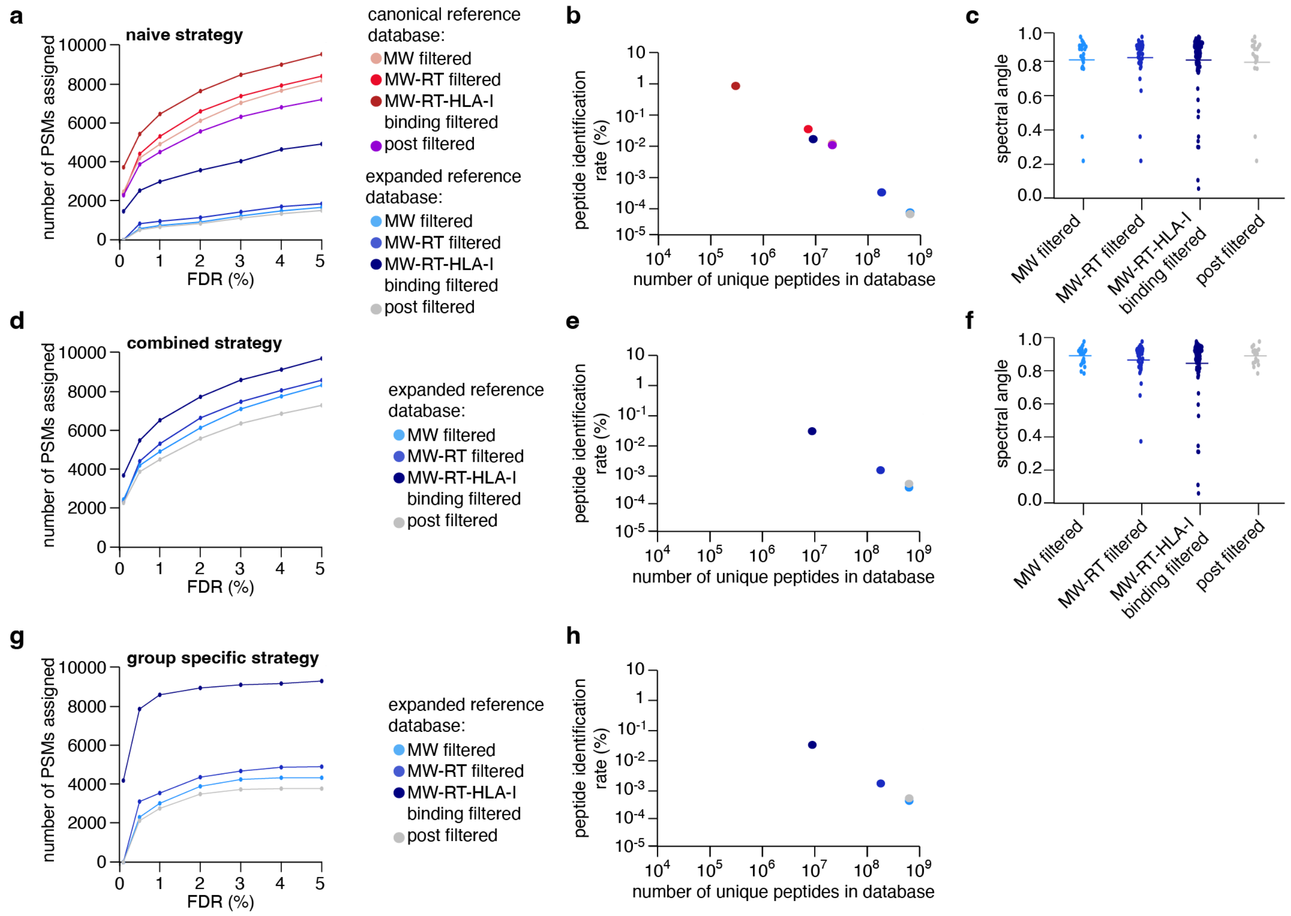

Supplementary Data Figure 9l Strategies for noncanonical peptide identification. (**a**-**h**) MSFragger search engine performance tested on K562-B*07-02 cell line immunopeptidomes, when confronted with a canonical reference database (RNA-informed CDS main ORF) or an expanded reference database (multiple RNA-seq informed strata together). Databases were either pre-filtered by MW-RT or pre- and post-filtered by MW-RT-HLA-I binding. Number of assigned peptide spectrum matches (PSMs) at given FDRs (**a**,**d**,**g**), identification rates (number of peptides identified at 1% FDR against database size) (**b**,**e**,**h**) and distributions of spectral angles for PSMs of noncanonical peptides identified at 1% FDR (**c**,**f**) using the naïve (**a**-**c**), combined (**d**-**f**), or group specific (**g**,**h**) strategy.

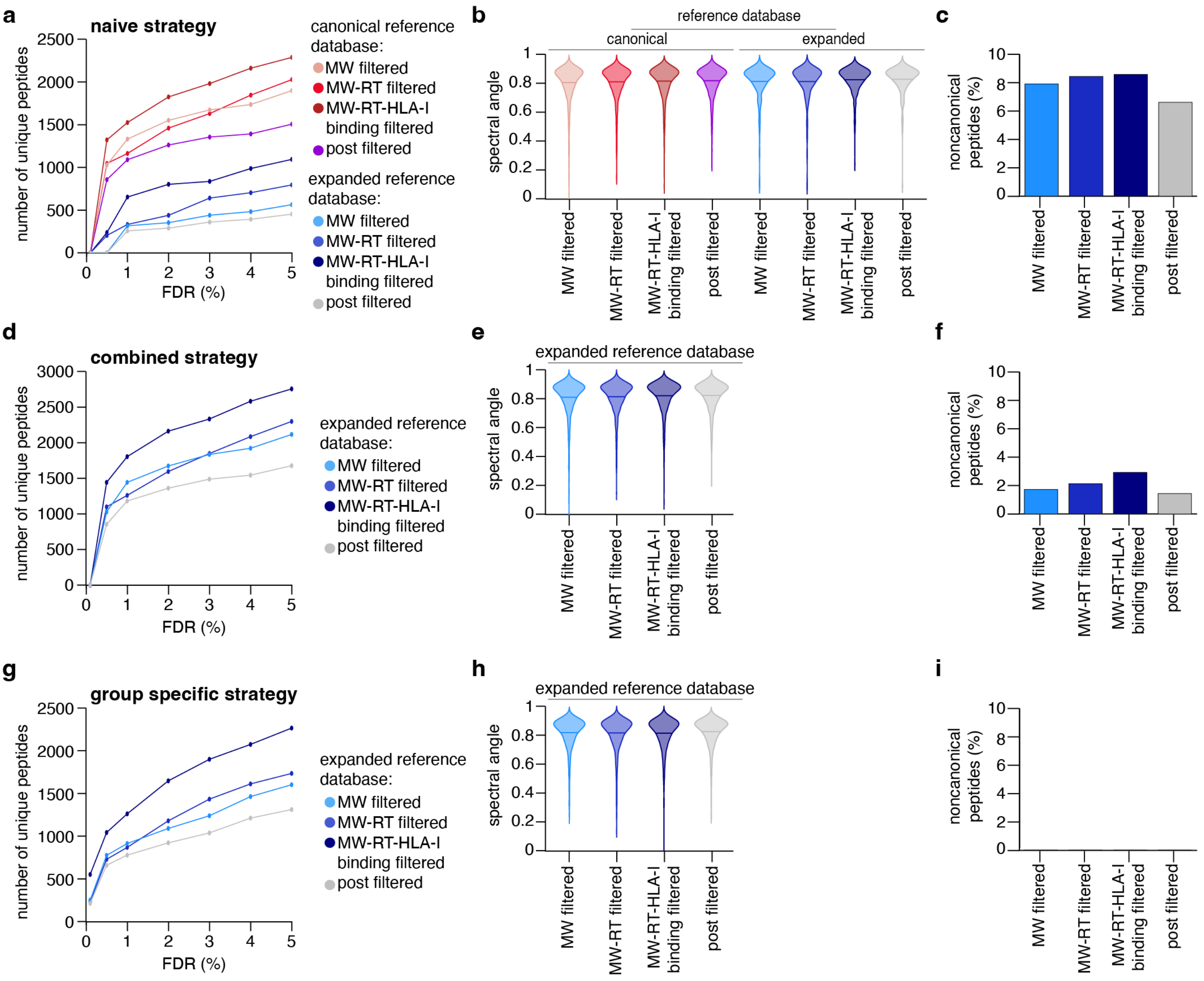

Supplementary Data Figure 10l Strategies for noncanonical peptide identification applied to the B721.221 cell line. (**a**-**i**) MSFragger search engine performance tested on B721.221-B*07-02 cell line immunopeptidomes, when confronted with a canonical reference database (RNA-seq informed CDS main ORF) or an expanded reference database (multiple RNA-seq informed strata together). Databases were either pre-filtered by MW-RT or pre- and post-filtered by MW-RT-HLA-I binding. Number of identified peptides at given FDRs (**a**,**d**,**g**), spectral angle distributions for PSMs of all peptides identified at 1% FDR (**b**,**e**,**h**) and percentages of noncanonical peptides identified at 1% FDR (**c**,**f**,**i**) using the naïve (**a**-**c**), combined (**d**-**f**), or group specific (**g**-**i**) strategy. The distribution of spectral angles is not shown for the group specific strategy as no noncanonical peptides were identified at 1% FDR using this strategy with any database filtering method.

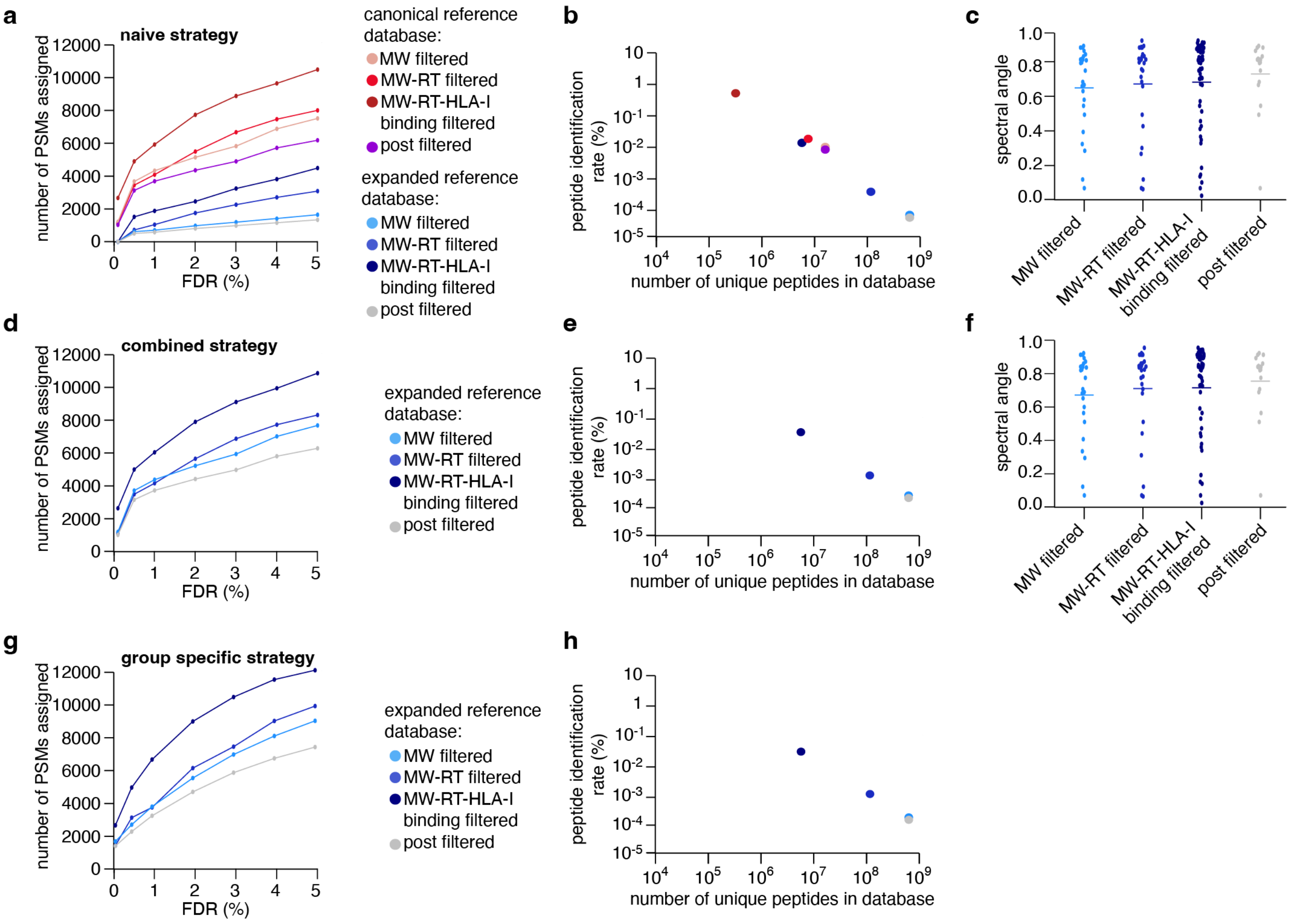

Supplementary Data Figure 11l Additional benchmarks for noncanonical peptide identification strategies. (**a**-**h**) MSFragger search engine performance tested on B721.221-B*07-02 cell line immunopeptidomes, when confronted with a canonical reference database (RNA-informed CDS main ORF) or an expanded reference database (multiple RNA-seq informed strata together). Databases were either pre-filtered by MW-RT or pre- and post-filtered by MW-RT-HLA-I binding. Number of assigned PSMs at given FDRs (**a**,**d**,**g**), identification rates (number of peptides identified at 1% FDR against database size) (**b**,**e**,**h**) and distributions of spectral angles for PSMs of noncanonical peptides identified at 1% FDR (**c**,**f**) using the naïve (**a**-**c**), combined (**d**-**f**), or group specific (**g**-**h**) strategy. The distribution of spectral angles is not shown for the group specific strategy as no noncanonical peptides were identified at 1% FDR using this strategy with any database filtering method.

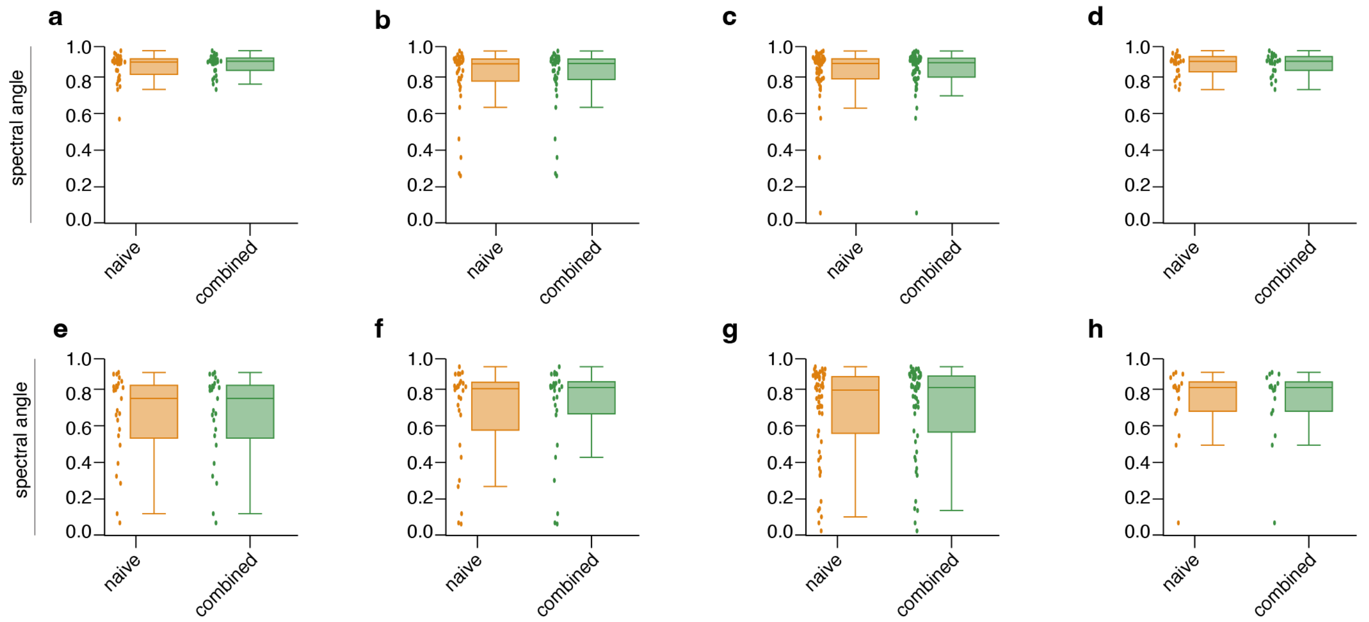

Supplementary Data Figure 12l Comparisons of noncanonical PSM quality for different FDR estimation methods. (**a**-**d**) Distribution of spectral angles of PSMs for noncanonical peptides identified at 1% FDR when analyzing K562-B*07-02 cell line immunopeptidomes using the MW- (**a**), MW-RT- (**b**), and MW-RT-HLA-I binding (**c**) pre-filtering of the database, as well as post-filtering (**d**) with different FDR estimation strategies. (**e**-**h**) Distribution of spectral angles of PSMs for noncanonical peptides identified at 1% FDR when analyzing B721.221-B*07-02 cell line immunopeptidomes using the MW- (**e**), MW-RT- (**f**), and MW-RT-HLA-I binding (**g**) pre-filtering of the database, as well as post-filtering (**h**) with different FDR estimation strategies.
